# Response to primary chemoradiotherapy of locally advanced oropharyngeal carcinoma is determined by the degree of cytotoxic T cell infiltration within tumor cell aggregates

**DOI:** 10.3389/fimmu.2023.1070203

**Published:** 2023-04-28

**Authors:** Maximilian Haist, Justus Kaufmann, Ivan-Maximiliano Kur, Stefanie Zimmer, Stephan Grabbe, Heinz Schmidberger, Andreas Weigert, Arnulf Mayer

**Affiliations:** ^1^ Department of Dermatology, University Medical Center of the Johannes-Gutenberg University, Mainz, Germany; ^2^ Department of Pathology, Stanford University School of Medicine, Stanford, CA, United States; ^3^ Department of Microbiology & Immunology, Stanford University School of Medicine, Stanford, CA, United States; ^4^ Department of Radiation Oncology and Radiotherapy, University Medical Center, Mainz, Germany; ^5^ Institute of Biochemistry I, Faculty of Medicine, Goethe-University Frankfurt, Frankfurt, Germany; ^6^ Institute of Pathology, University Medical Center of the Johannes Gutenberg University, Mainz, Germany

**Keywords:** head-and-neck cancer, oropharyngeal squamous cell carcinoma, spatial tumor biology, multiplex immunohistochemistry, CD8 T cells, tumor microenvironment, tumor stem cells, radiotherapy

## Abstract

**Background:**

Effective anti-tumor immune responses are mediated by T cells and require organized, spatially coordinated interactions within the tumor microenvironment (TME). Understanding coordinated T-cell-behavior and deciphering mechanisms of radiotherapy resistance mediated by tumor stem cells will advance risk stratification of oropharyngeal cancer (OPSCC) patients treated with primary chemoradiotherapy (RCTx).

**Methods:**

To determine the role of CD8 T cells (CTL) and tumor stem cells for response to RCTx, we employed multiplex immunofluorescence stains on pre-treatment biopsy specimens from 86 advanced OPSCC patients and correlated these quantitative data with clinical parameters. Multiplex stains were analyzed at the single-cell level using QuPath and spatial coordination of immune cells within the TME was explored using the R-package Spatstat.

**Results:**

Our observations demonstrate that a strong CTL-infiltration into the epithelial tumor compartment (HR for overall survival, OS: 0.35; p<0.001) and the expression of PD-L1 on CTL (HR: 0.36; p<0.001) were both associated with a significantly better response and survival upon RCTx. As expected, p16 expression was a strong predictor of improved OS (HR: 0.38; p=0.002) and correlated with overall CTL infiltration (r: 0.358, p<0.001). By contrast, tumor cell proliferative activity, expression of the tumor stem cell marker CD271 and overall CTL infiltration, regardless of the affected compartment, were not associated with response or survival.

**Conclusion:**

In this study, we could demonstrate the clinical relevance of the spatial organization and the phenotype of CD8 T cells within the TME. In particular, we found that the infiltration of CD8 T cells specifically into the tumor cell compartment was an independent predictive marker for response to chemoradiotherapy, which was strongly associated with p16 expression. Meanwhile, tumor cell proliferation and the expression of stem cell markers showed no independent prognostic effect for patients with primary RCTx and thus requires further study.

## 1 Introduction

Head and neck squamous cell carcinomas (HNSCC) represent the sixth most common cancer worldwide and are characterized by a high rate of local recurrence and metastatic dissemination (1). Traditional risk factors associated with HNSCC are tobacco and alcohol consumption. However, the past decades have revealed an increase of cases associated with high-risk human papillomavirus (HPV) infection, particularly for patients with oropharyngeal squamous cell carcinoma (OPSCC) which is now the most common type of head-neck cancer in many western countries. Patients with HPV-associated OPSCC tend to be younger, are more often non-smokers and demonstrate an improved survival compared to HPV-negative tumors that show worse outcomes and often present with primary resistance to existing treatments (2–4). Therefore, in the recent edition of the American Joint Committee on Cancer (AJCC) staging system HPV-positive and HPV-negative OPSCC were defined as separate entities with distinct molecular profiles and tumor characteristics (5). Owing to the morbidity associated with surgical resection, even with the most advanced robotic techniques, locally advanced cancers of the oropharynx are often treated by primary chemoradiotherapy (RCTx). Despite favorable prognosis for HPV-positive cases, treatment failures occur in both patient groups, and biomarkers that might improve risk stratification and personalized treatment approach are currently lacking. Basic research indicates that individual patient outcomes might be determined by a set of parameters, that involve:

(i) clinical tumor specifics, such as tumor stage and volume,
(ii) factors within the tumor microenvironment (TME) that confer resistance to existing treatments, such as radiotherapy-resistant tumor stem cells (6),
(iii) the coordination of effective anti-tumor immune responses within the TME.

The assumption that larger tumor volumes decrease the chance of cure by radiotherapy in HNSCC has been confirmed in many studies (7). Some conflicting data have been published on OPSCC, but larger and more recent series demonstrate its impact in this subregion (8–10). Tumor volume is also often measured as metabolic tumor volume, as assessed by PET-CT (11, 12). However, its clinical impact is predominantly observed for HPV-associated OPSCC (13).

There is a scarcity of data regarding stem cell density in OPSCC, and neither the exact phenotype of stem cells in this subregion of the head and neck nor the stem cell hierarchy has been elucidated (14). Prince et al. first showed evidence for CD44 as a stem cell marker (15). More recently it was observed that higher expression of CD44 predicted a poorer outcome in OPSCC treated with primary radiotherapy (RTx) (16). More specifically, Elkashti et al. (17) showed that CD271 defines a stem cell-enriched subpopulation of CD44 in HNSCC cell lines and human tissue specimens. These data led us to choose CD271 as a stem cell marker for the present study. Since the proliferative (“transit-amplifying”) compartment needs to be defined independently, we also included the proliferation marker Ki-67.

Intrinsic radiosensitivity is generally assumed to be higher in HPV-associated OPSCC, but convincing experimental evidence has only been presented recently (18, 19). These findings may in part explain the striking response to RTx observed in HPV-associated cancers in clinical studies (see above). While assessment of the HPV status is now standard for OPSCC, it was unknown for many of the older patients in our series with long-term follow-up, motivating us to include p16 in our antibody panel. Nuclear and cytoplasmatic p16 expression correlates precisely with HPV positivity and is suggested to be specific for HPV-positive OPSCC (20, 21).

Lastly, the recent success of anti-PD1 therapies for a subset of advanced HNSCC patients stressed the relevance of anti-tumor immunity in this tumor entity. In this regard, various authors reported an independent prognostic effect for CD3, CD4 or CD8 T cells in HPV-positive OPSCC patients (22, 23) demonstrating a significant role of anti-tumor immune responses not only for anti-PD1 therapies but for OPSCC tumor control and response to RCTx in general. Notably, distinct immune profiles have been observed in HPV-positive and negative HNSCC patients, suggesting that HPV might affect anti-tumor immune responses and immune checkpoint molecule expression (24). Also, it has been shown that expression levels of programmed cell death protein 1-ligand (PD-L1) might be a predictive biomarker for responses to anti-PD1/PD-L1 therapies (25). In particular, responses to PD-1 blocking antibodies were found to be significantly stronger for HNSCC patients with upregulation of tumor PD-L1 expression (26). In addition to the expression on tumor cells, PD-L1 is regularly expressed by immune cells. These include macrophages and NK cells (27, 28) that upregulate PD-L1 in response to IFN-γ release by effector T cells (29) and vice versa antigen presentation has been implicated in the upregulation of PD-L1 on effector T cells where PD-1/PD-L1 ligation has previously been implicated in immune exhaustion (30). As the PD-1/PD-L1 axis is a major immune checkpoint that confers adaptive immune resistance in various tumor contexts (25, 31, 32), we have employed CD8 and PD-L1 as surrogate markers of the anti-tumor immune response.

The advent of multiplexed *in-situ* imaging methods, such as multiplex immunofluorescence in combination with whole slide scanning (33) and single-cell segmentation now enables the quantitative exploration (34) of fundamental aspects of tumor pathophysiology and spatially profile complex biological systems at the single-cell level (35).

In the present study, we have employed multiplex immunofluorescence stains of pretherapeutic biopsy specimens from 86 OPSCC patients treated with primary RCTx on a dedicated tissue microarray to analyze the prognostic significance of the above mentioned biomarkers and investigate their interaction on a spatial level. We could demonstrate that p16 positive OPSCC patients had a better response and survival upon RCTx. Further, we observed a significant correlation between p16 expression levels and overall CD8 T cell (CTL) infiltration. While overall CTL infiltration was not associated with response or survival, we could show that the spatially organized infiltration of CTL in the tumor cell compartment was an independent predictive factor for subsequent response to RCTx. Moreover, we observed a favorable response to RCTx in patients with strong infiltration of PD-L1+ CTL. By contrast, we could not show an independent prognostic effect of the stem cell marker CD271 or the proliferation marker Ki67. If confirmed these findings have important implications for future stratification of patients for more or less aggressive treatment approaches.

## 2 Patients and methods

### 2.1 Patient cohort

**Figure 1:**
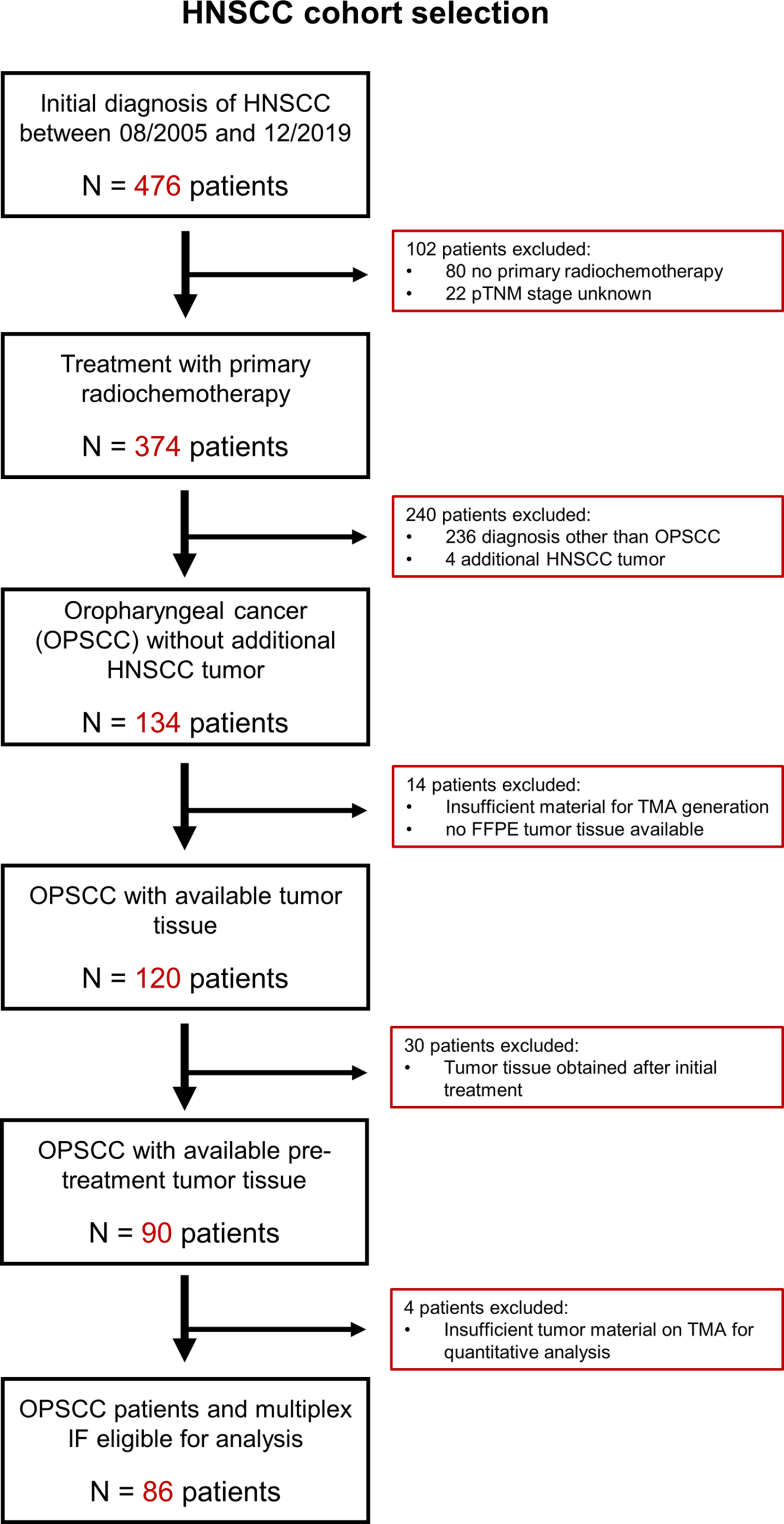
CONSORT diagram with criteria applied for the identification of OPSCC patients eligible for analysis. Overall, we identified 476 patients with diagnosis of head-neck cancer between August 2005 and December 2019. Thereof, we excluded 342 patients as they did not receive primary radiochemotherapy, have been diagnosed with a head-neck cancer other than oropharyngeal squamous cell carcinoma or had unknown pTNM stage. Also, we excluded 48 patients whose tissue samples did not meet the criteria for TMA generation and downstream image analysis. FFPE, formalin-fixed paraffin embedded; HNSCC, head-neck squamous cell carcinoma; IF, immunofluorescence; OPSCC, oropharyngeal squamous cell carcinoma; TMA, tissue microarray.

A cohort of 476 patients who were diagnosed with primary HNSCC between 2005 and 2019 at the University Medical Center Mainz, Germany, were screened. Eighty-six patients with survival follow-up data until April 2022 were retrospectively identified according to the following selection criteria (see **Figure 1**): Histopathological confirmed diagnosis of oropharyngeal squamous cell carcinoma (OPSCC), the absence of secondary HNSCC tumors diagnosed within the observation period, sufficient pre-treatment tumor tissue available for the generation of a tissue microarray (TMA), >30% of uncompromised tumor tissue on TMA for each patient, treatment with primary radiotherapy or chemoradiotherapy (RTx/RCTx), complete follow-up documentation of treatment outcomes including best overall response (BOR), progression-free survival (PFS) and overall survival (OS) (for details see **Supplementary Table 1**). Details on clinical-pathological parameters at initial diagnosis, subsequent treatments and survival data were collected from the patient´s medical records.

### 2.2 Generation of tissue microarray

Of the entire cohort of 476 patients, all 86 patients who met the selection criteria and for whom one or more paraffin tissue blocks were available were included in the present study. Formalin-fixed paraffin-embedded tissue blocks of these 86 OPSCC patients were retrieved from the archives of the Institute of Pathology. The area of malignancy was marked by a board-certified pathologist (S.Z.). The tissue microarray was constructed from 1,2mm diameter cores that were punched from a representative region of the tumor tissue blocks according to standard procedures. Subsequently, the 3µm thick tumor sections were stained with hematoxylin and eosin and slides were digitized using a whole-slide scanner (see **Supplementary Figure 2**).

### 2.3 Multiplex immunofluorescence staining

Seven-color multiplex fluorescence staining of the TMA for the antigens pan-Cytokeratin (CK) AE1/AE3, p16INK4A, CD271, PD-L1, Ki67 and CD8 was performed using the Opal Polaris 7-Color Manual IHC Detection Kit according to the manufacturer´s instructions as described previously (36). In brief, after cutting 3µm thick sections with high precision microtomes, specimens were incubated at 60°C for one hour and deparaffinized in a descending alcohol series. Pretreatment for multiplex immunofluorescence (mIF) was carried out using antigen-demasking buffers specific for the chosen antigen in each staining round. The subsequent staining process was performed six times in a serial fashion. Sections were incubated with the antibody diluent for 10 min at room temperature, followed by incubation with the primary antibody either for 60 min at 29°C or overnight at 4°C. After applying Opal polymer horseradish peroxidase-conjugated secondary antibody and Opal fluorophore solution each for 10 min, antibodies were removed by microwave treatment (heat-induced epitope retrieval; HIER) before a further round of staining. Finally, the nuclei were counterstained with DAPI and after rinsing with PBS, samples were covered with a coverslip using a fluorescence mounting medium. The antibodies, their dilutions, the according retrieval buffers as well as the sequence of usage are described in **Supplementary Tables 2**, **3**. The seven-color Opal slides were visualized using the Vectra Polaris Automated quantitative Pathology Imaging System. Spectral unmixing was applied to distinguish the seven different fluorescence signals.

### 2.4 Antibody screening, validation, and titration for multiplex immunofluorescence staining

The antibodies applied for mIF were first screened and validated using uniplex IF stains on tonsil tissue and OPSCC tumor tissue, with cross-validation and antibody titration by manual DAB-IHC (see **Supplementary Figure 1**). All validation was performed under the supervision of a board-certified pathologist (S.Z.) and collated with known expression patterns published online (The Human Protein Atlas, Pathology Outlines), as well as the published literature.

Chromogen-based IHC on tonsil and OPSCC tissues was carried out according to a standard procedure as published previously (37). Preparatory steps including HIER were carried out as outlined for multiplex IF staining (2.3.). Activity of endogenous peroxidase was blocked via incubation with 3% H_2_O_2_ in PBS for 5min. After subsequent washing steps, slides were incubated for 20min with normal horse serum 2,5% to block non-specific binding. Antibodies were diluted in PBS and sections were stained for 1h in a sealed humidity chamber at 29°C. After staining, slides were washed prior to incubation for 1.5h at 27°C with an HRP-conjugated polymer detection reagent, which was followed by another washing step. Bound antibodies were visualized using DAB substrate according to the manufacturer´s instructions. Sections were counterstained with hematoxylin, followed by dehydration, mounting, and imaging in brightfield mode on a slide scanner.

After chromogen-based IHC was used for all targets (panCK, p16, CD8, PDL1, CD271, Ki67), uniplex IF was conducted to optimize the antibody titrations, to generate spectral libraries required for multiplex IF analysis, determine the antibody-OPAL-dye pairs used for multiplex IF and their optimal staining sequence. Briefly, after deparaffinization and fixation, 3 µm tissue sections were processed with retrieval buffers for 15 min in a microwave oven. Similar to multiplex IF, sections were then incubated with the protein block followed by incubation with the primary antibody for 60 min at 29°C and after the application of the Opal polymer HRP secondary antibody and Opal fluorophore solution each for 10 min, slides were washed and counterstained with DAPI. In order to determine the optimal staining sequence for multiplex IF each uniplex IF was conducted three times with various HIER. In particular, for each antibody we stained three uniplex IF with 1x, 3x and 5x of heat-pre-treatments. Similar to IHC staining, the correct titration of the single antibodies in uniplex IF stains was chosen carefully to obtain a uniform, specific, and correct staining pattern (for details see **Supplementary Figure 1**).

### 2.5 Quantitative analyses

**Figure 2:**
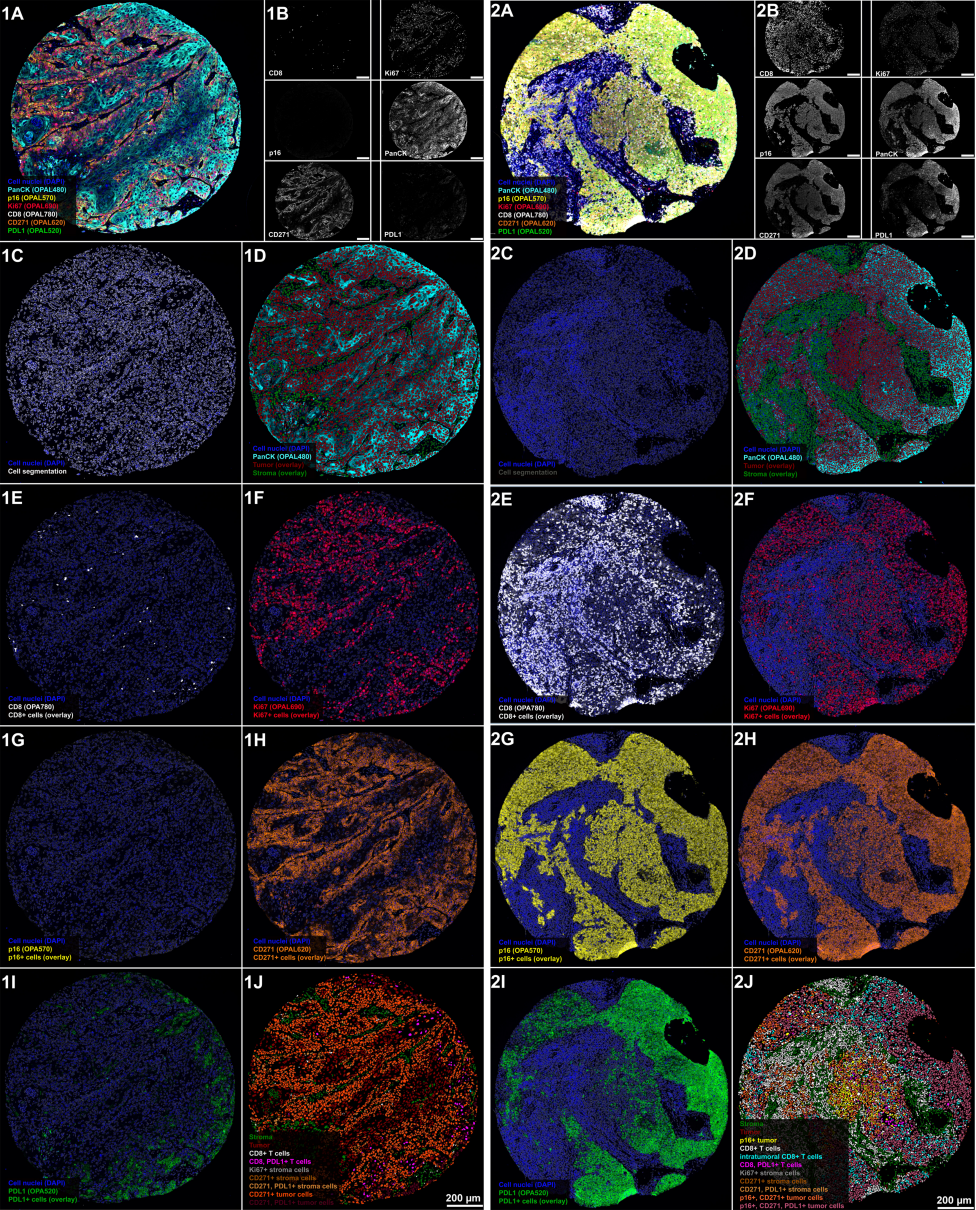
Stepwise procedure for the classification of cells in OPSCC tumor microenvironment in a representative example of a p16 negative (1) and a p16 positive OPSCC (2). **(A)** Original scanned 7-plex-immunofluorescence image (3.5x). **(B)** Gray color fluorescence images for each of the investigated markers (CD8, OPAL780; Ki67, OPAL690; p16, OPAL570; pan-cytokeratin, OPAL480; CD271, OPAL620 and PD-L1, OPAL520). Tumor core in 1A shows a p16-negative tumor with sparse infiltration by CD8 positive T cells, while tumor cells are predominantly positive for the proliferation marker Ki67 and the tumor stem cell marker CD271. Staining patterns of Ki67 and CD271 reveal a substantial co-expression of both markers in this tumor specimen. PD-L1 expression, by contrast, is sparse in this tissue sample and confined to tumor tissue. **(C)** Single-cell-based analyses were carried out using segmented nuclei (white border) as the starting point for cell detection in the open-source software QuPath. This approach enabled the systematic subclassification of all cell events. First, we subclassified cells as “tumor” or “stroma” based on the expression of pan-cytokeratin using QuPath´s machine learning features and user-defined examples (**D** tumor cells with a red border; stroma cells with an olive-green border). Second, we further subclassified cells using either intensity-thresholds for antibodies with a nuclear staining pattern (Ki67, **F** and p16, **G**) or an object-based classification algorithm for markers with a predominantly membranous staining pattern (CD8, **E**, CD271, **H**, and PD-L1, **I**), color-coding all cells according to the expression of the investigated markers. The resulting classification overlay, as shown in **(J)**, shows the final results of this classification approach focused on the major cell phenotypes.

Single‐cell‐based analyses were carried out for all TMA cores with a preserved tumor tissue >30% using the DAPI channel (blue) for the segmentation of cell nuclei in the open-source whole slide image analysis software QuPath as described previously (38) [https://qupath.github.io/ (34)]. Segmentation was followed by a stepwise procedure of further subclassification of cells (**Figure 2**). First, cells were classified as “tumor” or “stroma” based on the epithelial cell marker panCK and user‐defined examples for training of QuPath’s machine learning features, which additionally takes into account shape features of single-cells. Second, tumor and stromal cells were further subclassified using two different approaches: Cells expression markers with a nuclear staining pattern (Ki67 and p16) were identified using intensity thresholds in the relevant fluorescence channels. By contrast, markers with a membranous staining pattern (CD8, CD271, and PD-L1) were identified using an object-based training algorithm. Two independent observers, blinded to the patients´ survival data, conducted the quantification analysis and classified the respective cell types in relation to all nucleated cells per sample. This subclassification approach allowed a rigid assignment of markers to the investigated cells and thus a comprehensive phenotypic characterization of cells within the OPSCC TME. Quantitative data were finally correlated with clinical patient data. Markers used for the quantification of cell phenotypes are listed in **Supplementary Table 4** and the raw images and classification results are provided in **Supplementary Figure 3**.

### 2.6 Spatial analysis

Spatial analyses were carried out using the R-package “Spatstat”. Data generated from QuPath was imported and point patterns were constructed using the coordinates of cell centroids. The subclassifications computed in QuPath were applied as masks. Tumor cells within a distance of less than 35 µm of a stroma cell were classified as being part of the tumor-stroma-interface. Using a custom written R-function, CTL were subclassified into intratumoral CTL, stromal CTL and CTL at the tumor-stroma-interface (excluded infiltrate). The classification was based on the nearest distance of a CTL to both tumor and stroma. CTL within a 70 µm thick band at the tumor-stroma-interface were defined as excluded infiltrate. Spatial interaction analyses were performed using the inhomogeneous Cross-versions of Ripley’s K-function, the pair correlation function (pcf) and the nearest neighbor function (G-function). The resulting graphs were analyzed visually, and interactions were classified as (1) colocalization, (2) no interaction, (3) avoidance, (4) no analysis possible due to the limited size of at least one cell population. Spatial interaction was analyzed for different hypotheses. Cross-functions were calculated with a global confidence interval for the interaction of both marker-positive and marker-negative tissue populations. These were compared, and spatial interaction was deemed significant if there was any point, where confidence intervals did not overlap.

### 2.7 Statistical analysis

Descriptive statistics were used to analyze the baseline characteristics of the study population. Chi-square test was used to assess the association between the event of tumor relapse or disease progression and the quantitative data obtained from multiplex IF staining. Clopper–Pearson method was used to calculate 95% confidence intervals (CI) for the categorical variables. Testing for equality between patients with p16-positive *vs.* p16-negative tumors was performed using student’s t-test, Mann-Whitney test or Chi-square test. Moreover, the statistical analysis included Pearson´s and Spearman’s correlation analysis to test for correlations between continuous variables.

As a primary time-to-event endpoint, this retrospective cohort study used OS, which was estimated using the Kaplan–Meier product-limit method and log-rank statistics in R (39). PFS defined as secondary time-to-event endpoint of this study, was similarly estimated using the Kaplan-Meier product limit method and log-rank test. The association between BOR and the quantitative multiplex IF data was analyzed using Chi-square test.

The median duration of follow-up was calculated using the reverse Kaplan–Meier method. Independent prognostic values of the quantitative data obtained from multiplex IF and additional clinical patients´ characteristics were estimated using univariate and multivariate Cox proportional hazard models. Here, hazard ratios (HR) were provided with 95% confidence intervals (CI). Multivariate analysis was calculated for the significant (p ≤ 0.05) variables by the univariate test or *a priori* selection for biological relevance to evaluate their conjoint, independent effects on OS. In all cases, two-tailed p-values were calculated and considered significant with value of p < 0.05. SPSS (version 27, IBM, Ehningen, Germany), R (Version 4.0.3) and RStudio (Version 1.3.1093), and GraphPad PRISM (Version 9, San Diego, USA) were used for all analyses.

## 3 Results

### 3.1 Patient characteristics, response to chemoradiotherapy, and survival outcomes

A total of 86 patients have been treated with primary chemoradiotherapy (RCTx) for advanced OPSCC at the Department of Radiation Oncology of the UM Mainz between 2005 and 2019 with follow-up until 05/2022. Detailed patient characteristics are given in **Table 1**.

**Table 1:** Baseline patient characteristics.

N (%)	All patients	p16 positive OPSCC	p16 negative OPSCC	*p*-value
Overall number of patients	86	33	53	
Baseline patient and tumor characteristics				
Gender				0.341
Male	59 (68.6%)	25 (75.8%)	34 (64.2%)	
Female	27 (31.4%)	8 (25.0%)	19 (35.2%)	
Median age at initial diagnosis	61.0 yrs (45-85)	61.5 yrs (49-85)	61.0 yrs (46-82)	0.421
Tumor subtypes				0.716
- Oropharyngeal SCC	54 (62.1%)	19 (57.6%)	35 (66.0%)	
- SCC of the tonsil	8 (9.2%)	3 (9.1%)	5 (9.4%)	
- SCC of the soft palate	2 (2.3%)	1 (3.0%)	1 (1.9%)	
- SCC of the tongue basis	22 (25.3%)	10 (30.3%)	12 (22.6%)	
Grading >G2	30 (34.9%)	14 (42.4%)	16 (30.2%)	0.352
T-stage (>T2)	71 (82.6%)	26 (78.8%)	45 (84.9%)	0.562
N-stage (>N1)	73 (84.9%)	26 (78.8%)	47 (88.7%)	0.232
Cigarette smoking	63 (73.3%)	21 (63.6%)	42 (79.2%)	0.136
Cigarette smoking during treatment	25 (30.9%)	7 (13.2%)	18 (37.5%)	0.146
Amount smoking consumption (PY)	30.0 (0-160)	12.5 (0-160)	38.5 (0-100)	0.08
Alcohol consumption	51 (59.3%)	16 (48.5%)	35 (66.0%)	0.12
Alcohol consumption during treatment	10 (12.5%)	4 (12.1%)	6 (12.0%)	0.706
Heavy alcohol consumption	17 (19.8%)	7 (21.2%)	10 (18.9%)	0.792
Secondary cancer other than HNSCC^1^	19 (22.1%)	5 (15.2%)	14 (26.4%)	0.289
Treatment and response				
Induction CTx	42 (48.8%)	16 (48.5%)	26 (49.1%)	1
Induction CTx cycles	1.0 (0-3)	0.5 (0-3)	0 (0-1)	0.958
Discontinuation of induction CTx	5 (5.8%)	3 (9.1%)	2 (3.8%)	0.367
Neck dissection	26 (29.9%)	10 (30.3%)	16 (30.1%)	1
Concomitant systemic treatment	70 (80.7%)	27 (84.4%)	43 (79.6%)	0.335
Concomitant chemotherapy	55 (64.0%)	19 (69.4%)	36 (67.9%)	
- Cisplatin/5-FU	24	10	14	
- Cisplatin only	30	10	20	
- Cisplatin/5-FU/Cetuximab	1	0	1	
Cetuximab	15 (17.4%)	8 (25.0%)	7 (13.2%)	
Discontinuation of concomitant CTx	14 (16.3%)	7 (21.2%)	7 (13.2%)	0.376
Dose of initial RTx	70Gy (28.5-70.0Gy)	70Gy (36.0-70.0Gy)	70Gy (28.5-70.0Gy)	0.722
Duration of RTx	35 days (10-38)	35 days (10-38)	35 days (14-38)	0.405
RTx modification	15 (17.5%)			0.435
- Discontinuation of RTx	1 (1.2%)	1 (3.0%)	0	
- Dose reduction of RTx	14 (16.3%)	6 (18.1%)	8 (15.1%)	
Remission upon RTx	43 (58.9%)	23 (69.7%)	20 (37.7%)	**0.015**
Best-overall response				**0.012**
- Progressive disease	32 (37.2%)	7 (21.9%)	25 (47.2%)	
- Stable disease	1 (1.2%)	1 (3.0%)	0	
- Partial remission	6 (7.0%)	2 (6.1%)	4 (7.5%)	
- Complete remission	43 (50.0%)	23 (69.7%)	20 (37.7%)	
- N/A	4 (4.7%)	0	4 (7.5%)	
Survival data				
Median progression-free survival (95% CI)	14.0 months (0-35.9)	134.0 months (21.2-246.8)	6.0 months (1.2-8.4)	**<0.001**
Median overall survival (95% CI)	34.0 months (22.2-45.8)	136.0 months (22.6-249.4)	20.0 months (5.1-34.9)	**0.001**
Relapse of primary tumor	22 (25.3%)	4 (12.1%)	18 (34.0%)	**0.012**
Tumor progression	45 (65.9%)	8 (24.2%)	29 (54.7%)	**0.003**
Deceased	54 (62.8%)	14 (42.4%)	40 (75.5%)	**0.003**
Median follow-up time (95% CI)	70.0 months (64.3-75.7)	70.0 months (52.2-87.8)	71.0 months (64.1-77.9)	0.972

^1^ = secondary malignancies include cancers other than secondary tumors of the head-and-neck and pre-cancer lesions such as basalioma or carcinoma-in-situ of the cervix. The p-value is indicated in bold in case of significant differences between p16-positive and p16-negative patient sub-groups. 5-FU, 5-Fluoruracil; CI, confidence interval; CTx, chemotherapy; HNSCC, head-and-neck squamous cell carcinoma; PY, pack years; RTx, radiotherapy.

As HPV-positive OPSCC patients regularly show a better response and prognosis upon RCTx the patient cohort was divided by the p16 status as determined by review of IHC stains. Both patient cohorts showed comparable baseline characteristics, treatment modalities and follow-up periods, which allowed for the subsequent comparison of response and survival outcomes. Here, we found that patients with p16-positive OPSCC showed a significantly better response to primary RCTx (overall response rate: 75.8% *vs.* 46.2%; p = 0.012) with 69.7% of patients being disease-free after completion of primary RCTx (p=0.015). In accordance median PFS (134.0 months *vs.* 6.0 months, p<0.001) and median OS (136.0 months *vs.* 20.0, p= 0.001) were substantially better for p16 positive OPSCC patients (see **Table 1**).

### 3.2 Smoking during ongoing treatment is associated with an adverse survival outcome following chemoradiotherapy

**Figure 3:**
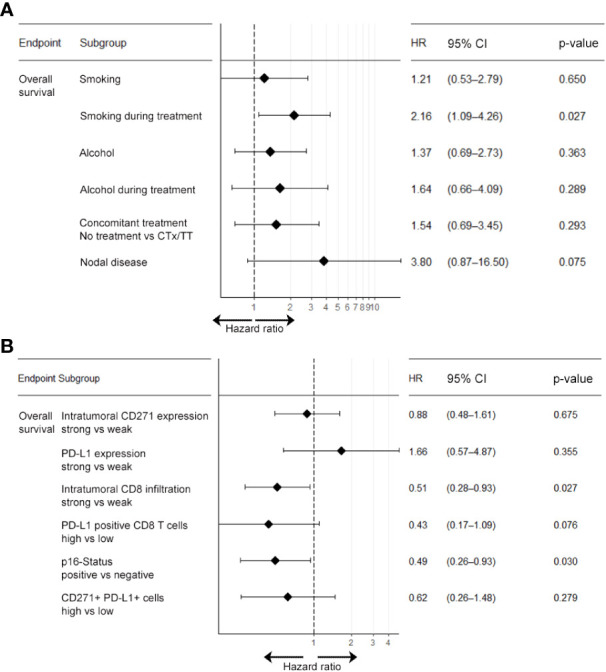
Multivariable Cox-regression analysis of clinical and molecular parameters associated with overall survival. Multivariate analysis for the endpoint overall survivals was carried out separately for clinical parameters **(A)** and biomarker parameters **(B)**. Results show that patients who smoked during treatment were at higher risk of shorter overall survival compared to non-smokers (HR: 2.16; 95% CI: 1.09-4.26). Also, patients with nodal disease at baseline were at higher risk of shorter overall survival (HR: 3.80; p=0.075), albeit this association was below statistical significance. CTx, chemotherapy; HR, hazard ratio; TT, molecular targeted therapy; 95% CI, 95% confidence interval.

Next, we investigated the relevance of clinical parameters for patient survival outcomes following chemoradiotherapy using univariate Cox-regression analysis. Given the established evidence in the literature that smoking and alcohol consumption reduce the efficacy of radiotherapy treatment and that lymph node metastases negatively affect overall prognosis we included these parameters into our analysis (40). In this univariable Cox-regression analysis, we observed that smoking, alcohol consumption, nodal disease, concomitant systemic treatment with either chemotherapy (CTx) or targeted therapy (TT) with Cetuximab during RTx and the BOR to RTx were significantly associated with overall survival. In particular, we observed a significant adverse effect of smoking and alcohol consumption during treatment in our patient cohort (see **Table 2**). This effect was confirmed in a multivariate Cox-regression model. Here, smoking during treatment (HR: 2.16, 95% CI: 1.09-4.26, p= 0.027) showed a particularly adverse effect on OS (see **Figure 3A**). By contrast, age at initial diagnosis, gender, and grading of the initial tumor did not show a significant prognostic value.

**Table 2:** Univariable Cox-regression analysis for overall survival stratified by clinical-pathological parameters and biomarker variables.

Parameters	Subgroups	HR	95%CI	p-value
A Clinical-pathological parameters				
Age (years)	>61 *vs.* ≤61	0.78	0.46-1.35	0.39
Gender	Male *vs.* female	1.19	0.66-2.15	0.556
Grading	≤G2 *vs.* >G2	0.58	0.31-1.05	0.07
Smoking	Yes *vs.* no	2.51	1.25-5.0	**0.009**
Smoking during treatment	Yes *vs.* no	3.31	1.88-5.86	**<0.001**
Smoking quantity	>30PY *vs.* ≤30 PY	2.3	1.26-4.21	**0.007**
Alcohol	Yes *vs.* no	2.21	1.21-3.99	**0.008**
Alcohol during treatment	Yes *vs.* no	2.99	1.42-6.30	**0.004**
Alcohol quantity	Heavy *vs.* moderate	2.77	1.43-5.35	**0.002**
T-stage	>T2 *vs.* ≤T2	1.41	0.63-3.13	0.401
N-stage	>N1 *vs.* ≤N1	2.82	1.01-7.81	**0.047**
Neck dissection	Yes *vs.* no	0.95	0.53-1.69	0.851
Concomitant treatment	CTx or TT *vs.* no treatment	0.52	0.27-0.99	**0.045**
BOR to RCTx	Response *vs.* no response	0.11	0.06-0.22	**<0.001**
B Biomarker parameters				
p16-status^1^	Positive *vs.* negative	0.38	0.21-0.70	**0.002**
Tumor volume^2^	High *vs.* low	0.53	0.31-0.92	**0.023**
CTL infiltration^2^	Strong *vs.* weak	0.74	0.43-1.28	0.28
CD271 expression^2^	Strong *vs.* weak	0.41	0.23-0.71	**0.002**
PD-L1 expression^2^	Strong *vs.* weak	0.52	0.30-0.90	**0.020**
Ki67 expression^2^	Strong *vs.* weak	0.75	0.43-1.29	0.29
Intratumoral CTL^2^	High *vs.* low	0.35	0.20-0.61	**<0.001**
Ki67+ tumor cells^2^	High *vs.* low	0.82	0.47-1.40	0.46
PD-L1 tumor cells^2^	High *vs.* low	1.05	0.61-1.79	0.87
CD271+ tumor cells^2^	High *vs.* low	0.65	0.38-1.12	0.11
CD271+ stroma cells^2^	High *vs.* low	0.48	0.28-0.84	**0.01**
PD-L1+ CTL^2^	High *vs.* low	0.36	0.21-0.64	**<0.001**
CD271, PD-L1 positive cells	High *vs.* low	0.39	0.22-0.67	**<0.001**

^1^ dichotomization was performed according to positivity or negativity of p16 staining in IHC; ^2^ groups were separated according to the median percentage of marker-feature positive cells within the patient cohort. The relative abundance of cell types that were characterized by more than a single variable (i.e., intratumoral CTL) was referred to the parent cell population (i.e., all CTL for intratumoral CTL or all tumor cells in case of Ki67 positive tumor cells). The p value is indicated in bold numbers when statistically significant. BOR, best overall response; HR, hazard ratio; CI, confidence interval; CTL, cytotoxic T lymphocytes; TT, targeted therapy; RCTx, radiochemotherapy.

### 3.3 Strong infiltration of CD8 T cells specifically into the tumor cell compartment and tumor-cell p16-positivity are associated with response to radiochemotherapy and favorable survival outcomes

In order to evaluate the prognostic value of the biomarkers investigated in our study, we correlated the quantitative data extracted from multiplex IF analysis with the clinical data from the patient cohort. Here, the medians of the investigated parameters were employed for subsequent dichotomization as we found that the investigated biomarkers showed no normal distribution as determined by Kolmogorov-Smirnov-Test.

**Figure 4:**
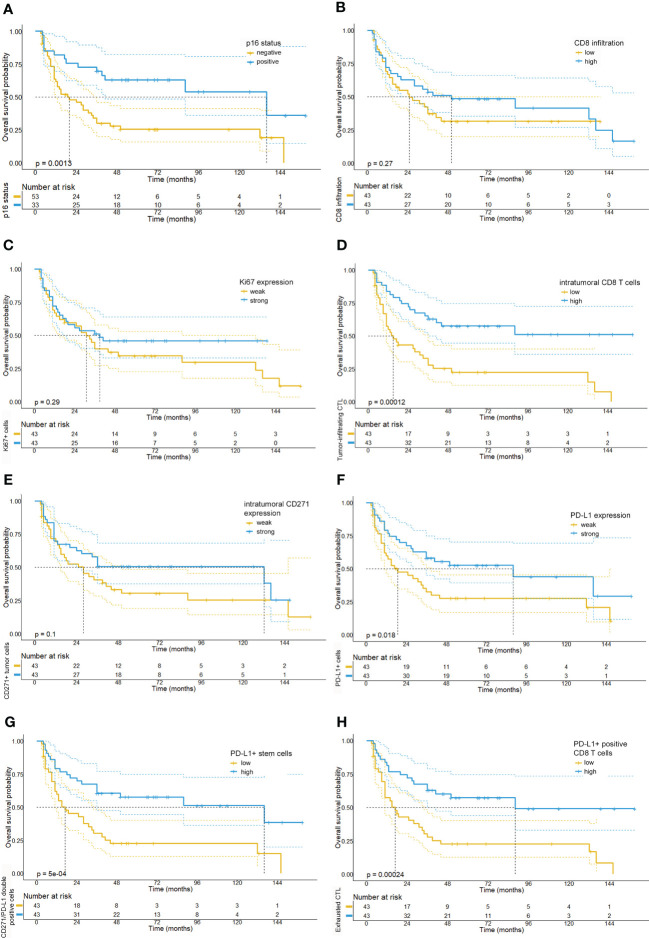
Kaplan-Meier survival plots depicting the overall survival probabilities of the investigated patient cohort stratified by biomarker expression levels. In accordance with the existing literature, we observed a favorable overall survival for patients with p16 positive tumors **(A)**. By contrast, neither overall CD8 T cell infiltration (median OS: 25 months *vs.* 50 months, p= 0.28) **(B)**, Ki67 expression (median OS: 31 months, 95% CI: 19.97-42.0 *vs.* 39 months, p= 0.287) **(C)**, or intratumoral CD271 expression (median OS: 28 months *vs.* 132 months, p=0.105) **(E)** were significantly correlated with overall survival. However, we detected that a intratumoral infiltration by CD8 T cells (median OS: 15 months *vs.* NR, p <0.001) **(D)** and a high expression of PD-L1 on CD8 T cells (median OS: 39 months *vs.* NR, p <0.001) **(H)** were significantly associated with a prolonged overall survival. Also, high levels of PD-L1 expression (median OS: 19 months, 95% CI: 3.5-34.5 *vs.* 88 months, 95% CI: 6.9-169.1, p=0.018) **(F)** and a high number of PD-L1/CD271+ double positive cells **(G)** were associated with a favorable prognosis in the investigated tissue cores (median OS: 17 months (5.4-28.6) *vs.* 136 months (21.9-250.1), p<0.001).

**Figure 5:**
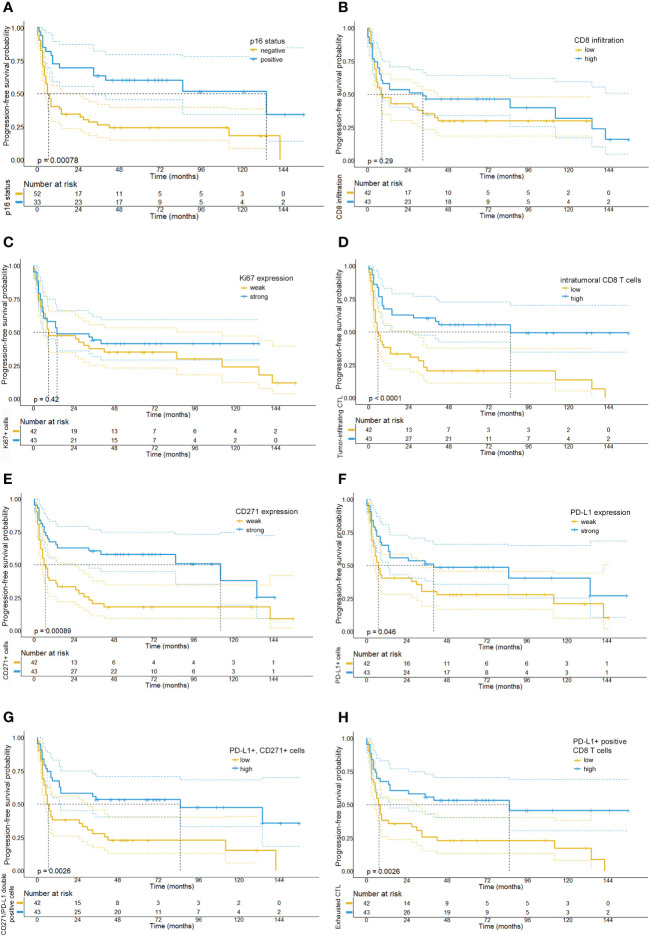
Kaplan-Meier survival plots depicting the progression-free survival probabilities of the investigated patient cohort stratified by biomarker expression levels. Our analysis shows that patients with p16-positive tumors **(A)**, a strong intratumoral CD8 T cell infiltration **(D)**, high PD-L1 expression levels **(F)** and low numbers of CD271+ tumor cells **(E)** had a prolonged progression-free survival. Moreover, we observed that high numbers of PD-L1 and CD271 double positive cells **(G)** and high numbers of PD-L1 positive CD8 T cells **(H)** were similarly associated with a longer PFS. By contrast, relative quantities of Ki67 **(C)** or CD8 T cell numbers **(B)** were not found to be associated with PFS.

**Figure 6:**
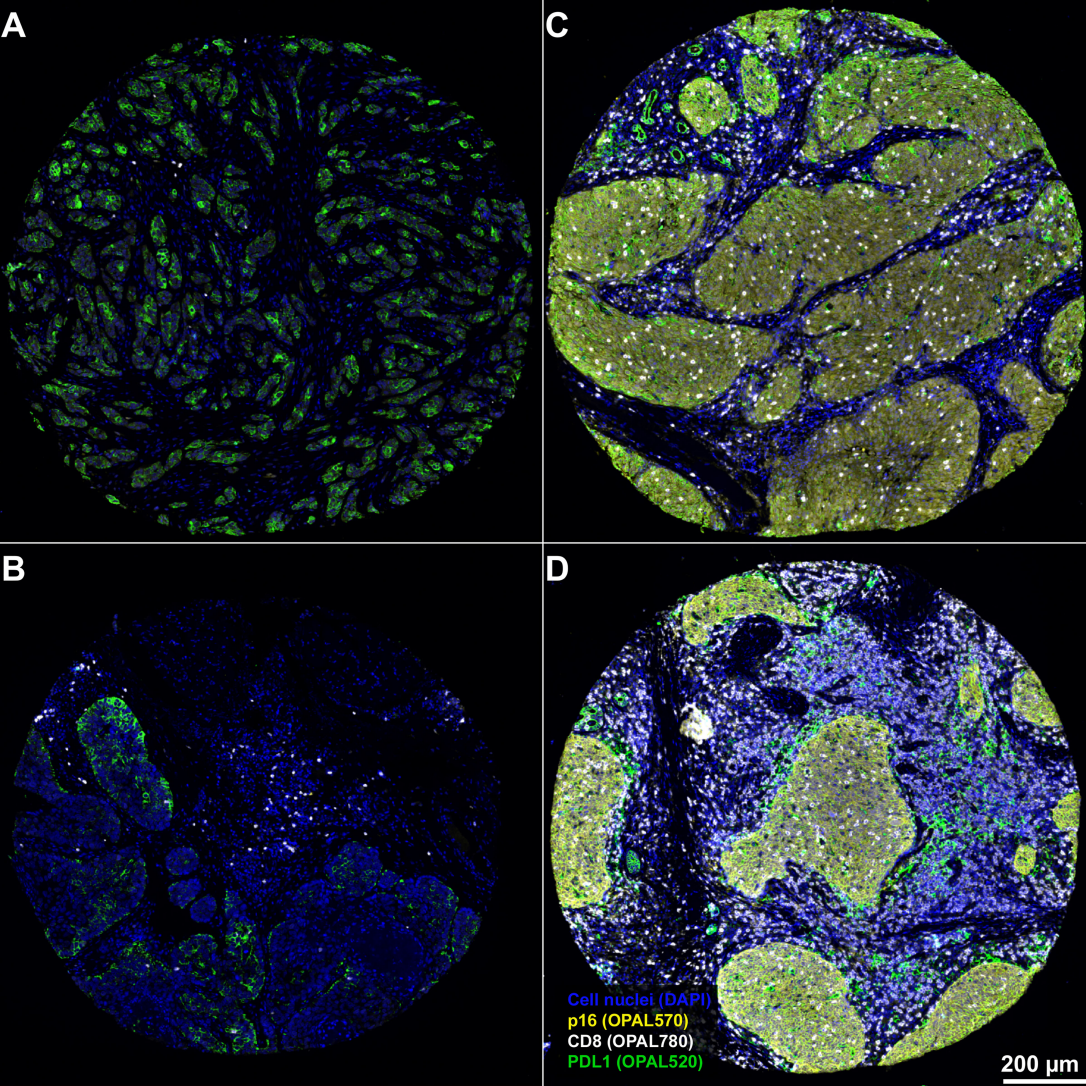
Representative examples from 4 different patients for the spatial distribution of CD8^+^ CTL within the OPSCC tumor microenvironment. Results demonstrates a weak infiltration of CD8 CTL within p16-negative tumors **(A, B)** and that CD8 CTL are predominantly clustered within the tumor stroma of p16-negative patients. By contrast, there is a strong tumor-infiltration by CD8 CTL in p16 positive tumors **(C, D)**. The PD-L1 expression in p16 negative tumors is predominantly confined to tumor cells, while PD-L1 expression in both p16 positive tumors is relatively heterogeneously distributed between tumor, stroma and CD8 CTL. Magnification, 3.6x. Scale bar in D applies to all panels of the figure.

In line with the existing literature our analysis confirmed a favorable survival outcome in p16 positive OPSCC patients. Also, we observed that patients with an overall high expression of PD-L1 and a strong tumor-infiltration by CTL showed a significantly better OS, PFS and response upon RCTx as compared to patients with expression below the median (see **Figures 4**, **5**, **Supplementary Figures 5–7**, **Supplementary Tables 7**, **8**, as well as **Table 2**). By contrast, the expression of CD271 within the tumor cell compartment was not prognostic. These observations were confirmed in a multivariate Cox-regression model which showed a favorable effect on OS and PFS for patients with a stronger tumor-infiltration by CTL (see **Figures 3B**, **4**–**6** and **Supplementary Figure 6**).

By contrast, the expression of the proliferation marker Ki67 or the intratumoral PD-L1 expression were not significantly associated with either survival outcome. Notably, the quantitative data among the p16 positive and p16 negative patient cohorts were relatively equally distributed, with the exception of a significantly stronger expression of CD271 and a stronger infiltration by CTL in p16-positive OPSCC (see **Supplementary Table 5**). In this regard, we also observed a strong correlation between p16 expression and the expression levels of CTL (r: 0.358, p<0.001) and CD271+ cells (r: 0.414, p<0.001) in the overall patient cohort (see **Supplementary Table 6**).

### 3.4 Spatial distribution and phenotype of CD8 T-cells is predictive for response and survival upon chemoradiotherapy

Due to the significant role of anti-tumor immune responses for durable response to RCTx we further investigated the role of CTL in our OPSCC cohort. Here, our analysis unveiled a significant prognostic role of the spatial distribution and the phenotype of CTL. For most of the tumor tissues examined, we found a relatively sparse infiltration by CTL (median: 5.1%). Notably, CTL were visually arranged in a compartmentalized fashion, so that CTL regularly exhibited a pronounced clustering in the stroma and infiltrated the tumor cell compartment to varying degrees (see **Figure 6**). Strikingly, we observed that both the overall abundance of CTL (r: 0.358, p <0.001) and their infiltration into the tumor compartment (r: 0.339, p=0.001, see **Supplementary Table 6**) were significantly associated with p16 tumor positivity, whereas other clinical parameters such as smoking status or alcohol consumption were not associated with CTL infiltration.

With regard to response and survival of OPSCC patients, we found that the overall infiltration by CTL into the tumor, irrespective of the investigated compartment, was not associated with a longer OS (25 months *vs.* 50 months, p= 0.28) or PFS (8 months *vs.* 31.7 months, p=0.288). However, our data revealed that the relative abundance of CTL within the tumor compartment was significantly associated with response (p<0.001) and survival upon RCTx, indicating towards the prognostic role of the spatial CTL distribution (see **Supplementary Figures 4, 5**). In particular, patients whose tumor compartment contained a higher amount of CTL (median: 43.1%) showed a significantly longer median OS and PFS as compared to patients with a weak intratumoral CTL infiltrate (median OS: 15 months *vs.* NR, p <0.001 and median PFS: 5 months *vs.* 85 months, p<0.001) (see **Figure 3**).

In addition to the spatial distribution of CTL, our data unveiled a prognostic role of the CTL phenotype: In particular, a stronger expression of PD-L1 in CD8^+^ CTL was significantly associated with response (p=0.014, see **Supplementary Figure 5**) and survival upon RCTx (median OS: 17 months *vs.* 88 months, p<0.001 and median PFS: 7 months *vs.* 85 months, p=0.003) (see **Figures 4**–**6**).

### 3.5 Spatial distribution of CD8 T cells within the TME and abundance within the invasive tumor front of p16+ OPSCC predicts response to chemoradiotherapy

Previously 3 main patterns of immune infiltration have been described for various solid tumors, namely (1) immune desert, (2) excluded infiltrates and (3) inflamed tumors. This suggests an important role of the invasive tumor-stroma front, which is located at the tumor-stroma interface, for anti-tumor immune responses. Therefore, we classified CTL by their localization within the TME as either (1) intratumoral, (2) stromal or (3) excluded infiltrate (**Supplementary Figure 9**). A high ratio of CTL within the tumor cell compartment was correlated with a better OS and PFS in all tumors (median OS: 15 months *vs.* NR, p <0.0001 and median PFS: 5 months *vs.* 85 months, p<0.0001). This was true for p16-positive (median OS: 39 months *vs.* NR, p <0.01 and median PFS: 33 months *vs.* NR, p<0.01) as well as p16-negative tumors (median OS: 14 *vs.* 26 months, p=0.11 and median PFS: 4.5 *vs.* 8 months, p=0.11), albeit the correlation was below statistical significance in the p16-negative cohort when stratified by the median of tumor-infiltrating CD8 T cells (see **Supplementary Figure 11**). Using univariable Cox proportional hazard models with continuous predictors (non-thresholded) the abundance of tumor-infiltrating CD8 T cells did however significantly correlate with PFS (HR: 0.99, 95% CI: 0.98-1.0, p=0.026) and OS (HR: 0.98, 95% CI: 0.97-1.0, p=0.01) in the p16-negative patient cohort, while this did not apply for the overall CD8 infiltration. Furthermore, the number of CTL at the invasive tumor front was prognostic in p16+ tumors (median OS: 41 months *vs.* NR, p <0.015 and median PFS: 33 months *vs.* NR, p<0.01). This was particularly the case for PD-L1+ CTL (median OS: 39 months *vs.* NR, p <0.02 and median PFS: 33 months *vs.* NR, p<0.04) and Ki67+, PD-L1+ CTL (median OS: 41 months *vs.* NR, p <0.031). Additionally, we investigated CTL infiltration within the tumor-stroma interface in more detail using spatial analysis. Here, we found that a larger tumor-stroma interface was correlated with an increased ‘excluded infiltrate’ of CTL (r = 0.3; p<0.01) and negatively correlated with the amount of intratumoral CTL (r = -0.3; p<0.001). Furthermore, a strong PD-L1-expression within the tumor-stroma interface correlated with both intratumoral (r = 0.9; p< 0.001) and ‘excluded’ PD-L1+ CTL (r = 0.7; p<0.001). Last, we observed that the relative size of the tumor-stroma interface in relation to the total tumor volume correlated strongly with poorer outcomes and was significantly associated with disease progression (median OS: 13 months *vs.* 136, p <0.0001 and median PFS: 5 months *vs.* 85 months, p<0.0001) which was particularly found in p16-negative tumors (median OS: 15 months *vs.* 28 months, p <0.03 and median PFS: 6 months *vs.* 8 months, p<0.05).

### 3.6 Expression of CD271 is predominantly confined to the tumor cell compartment but was not associated with adverse survival outcomes

Due to the known role of CD271 as a stem cell marker and the proposed adverse effect on OPSCC patient prognosis and response to RTx, we further investigated the expression of CD271 in our OPSCC patient cohort. The expression of the stem cell marker CD271 was predominantly confined to the tumor compartment. We found CD271 expression particularly in the tumor of p16-positive patients to be widespread and intense with a median of 69.0% CD271+ tumor cells, while CD271 expression in p16 negative patients was much more heterogeneously distributed with expression in a median of 44.6% of tumor cells. By contrast, CD271 expression in the stroma was weak and mainly found on endothelial and mesenchymal cells (see **Supplementary Figure 8**). In most tumor samples the expression pattern of CD271 showed a significant co-localization with the proliferation marker Ki67 (see **Supplementary Figure 8**). However, this pattern was not uniformly found in all tumor samples and some patients showed a homogenous moderate or high CD271 expression in the tumor.

Dichotomization of the patient cohort based on the median of the percentage of CD271^high^ tumor cells (median: 63.2%) did not show statistically significant differences with regard to response (p=0.260) or survival for patients with a high fraction of CD271 positive cells (see **Figure 4**). Patients with a maximum of 63.2% of CD271^high^ tumor cells showed a median overall survival of 28 months, while patients with higher percentages of CD271^high^ cells had a median survival of 132 months (log-rank p = 0.105). This association was particularly confirmed in p16 positive patients, which might be inferred from the stronger proliferative properties of these tumors. Conflicting with these observations, stratification of patients according to the CD271 expression in the tumor revealed no significant impact on survival (p=0.105) or response to RCTx (p=0.260).

## 4 Discussion

The introduction of the novel AJCC TNM8 staging guidelines for OPSCC recognized the substantially better prognosis for HPV-positive compared to HPV-negative OPSCC patients which has been attributed to immunogenicity of viral proteins inducing strong anti-tumor immune responses and therefore reflects their distinct tumor biology. The emerging role of anti-tumor immunity for HPV-positive OPSCC resulted in the advent of novel immunotherapy treatments and attempts for treatment de-intensification that are currently pursued in clinical trials (41, 42). However, it remains a key challenge to identify both HPV^+^ and HPV^-^ OPSCC patients that are at high risk of primary treatment resistance and locoregional tumor recurrence and who therefore require more intensive treatment. Increasing evidence supports the role of the TME in cancer progression, but the understanding which mechanisms drive treatment resistance within the TME, in how far the crosstalk between neoplastic cells and immune cells, as well as their spatial location and functional orientation leads to tumor-rejecting or tumor-promoting environment remains incomplete (43, 44).

Several recent studies have characterized the role of tumor-infiltrating lymphocytes and their phenotype for response to OPSCC treatments and overall patient prognosis, but how the spatial organization within the immune TME might determine the effectiveness of subsequent RCTx has not yet been extensively investigated (4, 45–48).

Also, the inability of RCTx and anti-tumor immune responses to eradicate cancer stem cells is among the most supported theories to explain cancer treatment resistance, recurrence and metastasis (17).

To accurately identify markers for response to primary RCTx we have applied 7-plex mIF stains on pre-treatment OPC biopsies, a digital image analysis-framework based on single-cell segmentation and dedicated spatial statistics methods that allow for a detailed characterization of the OPC immune landscape.

Using this framework, we were able to show that the spatial organization and the phenotype of CTL is one mechanism mediating better response to RCTx in OPSCC patients. While favorable response and prognosis of HPV-positive patients is established in the literature and was introduced into AJCC staging system of OPSCC (2, 20, 49, 50), it has not been until more recently, that HPV status has been linked to anti-tumor immunity: In this regard, it has been shown that HPV-positive OPSCC are characterized by an enhanced immune cell infiltration (46), a T-cell inflamed tumor phenotype and distinct T-cell gene signatures (51, 52). Our results complement these findings, confirming a strong correlation between CTL infiltration and p16-expression levels. However, as opposed to previous reports, we were able to demonstrate for the first time that infiltration specifically of the epithelial-neoplastic compartment by CD8-positive cytotoxic T lymphocytes is the key marker for tumor sensitivity to primary (chemo-) radiotherapy in OPSCC. Remarkably, high levels of CTL within the tumor cell compartment may even evoke strong tumor responses in HPV-negative OPSCC patients. Together with multivariate regression analysis, these data strongly suggest that CTL phenotype and spatial organization rather than HPV status alone might impact the survival of OPSCC patients. Indeed, recent reports support our observation that CTL infiltration into the OPSCC TME is highly coordinated, as the authors identified distinct spatial features within the TME that were characterized by intertumoral aggregates of CTL, CD4 T cells and dendritic cells that formed a positive feedback loop via the local production of lymphoid cell attracting and organizing chemokines (53). Not least, our findings also indicate that pre-existing tumor T cells can survive radiation and mediate antitumor responses without the assistance from newly infiltrating T cells (54).

Second, we found PD-L1 expression to be widespread and intense for the majority of patients within our cohort (median percentage of PD-L1 positive tumor cells: 35.5%) using tumor clone E1L3N. In particular, among the 86 patients examined in our study, 79 (91.8%) expressed PD-L1 and all of these displayed cell surface staining of 5% or more of tumor cells (cut-off >5%). The differences to previous reports with regard to high PD-L1 expression levels might likely be inferred from the use of different Ab clones. Each Ab targets different PD-L1 epitopes with different affinity resulting in different staining patterns (55).

PD-L1 expression was mainly restricted to the tumor periphery (67.9%) at the interface between tumor cell nests and inflammatory stroma (see **Supplementary Figure 10**), whereas 32.1% showed diffuse PD-L1 expression throughout the tumor cell nests, which confirms previous observations (56). In contrast to other reports we did however not find a correlation of HPV-status and PD-L1 expression levels (median PD-L1 expression: 35.7% *vs.* 35.4%, p=0.737) (57), nor did we observe a statistically significant association with overall CTL infiltration (58), albeit there was a trend towards stronger CTL infiltration in patients with strong tumor PD-L1 expression.

Survival analysis revealed that a strong PD-L1 expression was related to better OS, PFS and response in univariate analysis, which is in accordance with others (4, 58, 59). These results were however not confirmed in our multivariate regression model.

The observation that patients who presented with a strong intratumoral PD-L1 expression and CTL-infiltration within the tumor cell compartment achieved better survival outcomes demonstrates that PD-L1 expression cannot be interpreted solely as a marker of immune evasion in the context of OPSCC but might rather reflect a response to activation of an endogenous inflammatory immune reaction at the tumor site (4, 60).

By contrast, there is conflicting data on the role of PD-L1 expression by T cells: On the one hand, Diskin and coworkers showed that T cell expression of PD-L1 maintained intra-tumor immune tolerance that resulted from the suppression of neighboring effector T cells via the PD-L1/PD-1 axis and the promotion of M2-macrophage polarization (30). This finding of PD-L1 expression on effector T cells driving immune tolerance was confirmed in the context of advanced melanoma and lung cancer where the abundance of PD-L1 positive CTL was indicative for an adverse outcome (31, 61). Similar to the role of PD-L1 for CD8 T cells, previous reports showed that PD-L1 signaling also induced a regulatory phenotype among memory CD4 T cells and promoted the conversion of CD4 T cells to regulatory T cells (32). In line with these observations, Zheng and coworkers described the occurrence of CD8+ regulatory T cells that expressed PD-L1 thereby suppressing CD8 T cell proliferation (31).

However, recent data suggested that PD-L1+ T cells might be a favorable prognostic factor in head-neck cancer patients (62). A potential explanation for these opposing functions of PD-L1 expression for T cell activity has recently been proposed by Bromberg and coworkers who showed that exclusive *in*-*cis*-binding of PD-L1 promotes anti-tumor immunity, while engagement of CD80 and PD-L1 *in-trans* inhibits immune-responses (63). Also, Mandal et al. previously reported that FoxP3+ Treg infiltration within p16-positive and p16-negative HNSCC tumors, was among the strongest for many cancer types, which suggests that HNSCC is characterized by both high levels of immune infiltration and high degrees of immunosuppression (64). This finding is reflected by our observation of a strong PD-L1 expression observed within the CD8 T cell compartment and stresses the importance of further investigation delineating the exact phenotype and functional role of these CD8+ T cells.

Third, our data indicate that the invasive tumor stroma front might be a critical compartment for orchestrating anti-tumor immune responses as previously suggested for colorectal carcinoma (65). In particular, we observed that HPV-negative patients which presented with larger tumor-stroma interface areas showed poorer outcomes as compared to HPV-negative OPSCC patients with a small tumor-stroma interface area and were infiltrated to a smaller degree by CTL. As a potential mechanism driving exclusion of CTL in tumors with large tumor-stroma interfaces we have identified a stronger PD-L1 expression (r=0.9; p<0.001) within the tumor stroma interface area that correlated with intratumoral CTL infiltration.

Regarding the role of the tumor stem cell niche our results have raised questions: While CD271 showed the expected expression predominantly within the tumor cell compartment, and only to minor parts in the tumor stroma, we observed (i) a strong heterogeneity in CD271 expression among the overall patient cohort and a stronger expression of CD271 among HPV-positive OPSCC. Also, we did not observe (ii) a spatial co-localization of CD271+ tumor cells with PD-L1 which would have been expected given previous observations from human triple-negative breast cancer and lung adenocarcinoma describing a strong correlation between tumor stemness and PD-L1 expression (66–68) and (iii) no impact on patient neither on response to RCTx or patient prognosis. It is unlikely that this finding is the result of the technical shortcomings of our multiplex immunofluorescence approach, given that Tran and coworkers previously reported similar expression patterns in HNSCC patient samples (17).

However, the findings of our study do not invalidate the concept of tumor-stem cell mediated tumor recurrence and progression. As shown previously, it might be necessary to use a set of markers that might accurately identify tumor stem cells and their distinct properties, such as resistance to chemotherapy (69), radiotherapy and oxidative stress (70), self-renewal and maintaining pluripotency (71). In this regard, it seems conceivable that the observed CD271 expression might not entirely be attributed to the stem cell character of tumor cells but might also be found in certain differentiated cells expressing nerve growth factor receptor.

We acknowledge several limitations of our study, including the retrospective and single-center nature, which may introduce a selection bias. Furthermore, the experimental technology and computational framework presented here show certain limitations: For example, tissue quality and poorly fixed tissues, as well as the presence of autofluorescent structures might affect imaging, result in misleading staining patterns and therefore introduce a bias into further downstream analysis. Furthermore, development of a 7-plex antibody panel requires extensive validation, which is a time-consuming and costly process. Here, available scoring systems working with clinically available H&E or DAB-IHC stains might be provide a more cost-efficient way to predict patient prognosis (41). Moreover, more comprehensive insights into the reciprocal relationship between the stem cell compartment, anti-tumor immune responses and other mechanisms that drive treatment resistance will be needed to better define patient prognosis and response to treatment. The currently emerging multiplexing technologies capable of assessing >50 functional marker proteins will be able to address some these questions and give more detailed insights into this spatial relationship. Also, spatial RNA-sequencing approaches will allow for a more precise characterization of the TME, delineate signaling mechanisms that drive immune cell infiltration and interaction and identify HPV integration into the host genome (1, 72, 73).

Last, we acknowledge that our investigation was entirely focused on patients given primary RCTx and did not include patients given additional immune-checkpoint-blocking therapies which are increasingly applied in many HNSCC patients.

In conclusion, our results provide a number of clinically well applicable baseline biomarkers, as well as patient baseline characteristics associated with response to primary RCTx in advanced OPSCC patients. On a functional level, we confirmed the critical role of spatial organization and the phenotype of CTL within the TME for response to primary RCTx. Our results also indicate that HPV-infection and anti-tumor immunity are closely interlinked and may in part explain the success of anti-PD1 therapies in HPV-positive OPSCC patients. By contrast, OPSCC proliferative activity and the number of CD271+ cells were not found to be prognostic. Given the strong heterogeneity within the tumor stem cell compartment our results will require further investigations taking into account phenotypical and functional diversity of tumor stem cells in OPSCC such as with the newly emerging multiplexing technologies that are able to characterize this compartment at a more refined level.

## Data availability statement

All data presented in this study can be found in the article and its supplementary data. Clinical and quantitative data, as well as single cell data tables that support the findings of this study are openly available at Dryad: https://datadryad.org/stash/share/e6h1WXezxMRbZRizEJ2nmViGxQtx1TCmEoTFJmN0jkg. Raw primary imaging data can be obtained from the authors directly upon reasonable request.

## Ethics statement

The studies involving human participants were reviewed and approved by local ethics committee (Ethik-Komission der Landesärztekammer Rheinland-Pfalz, No: 837.150.14 (9389-F)). The patients/participants provided their written informed consent to participate in this study.

## Author contributions

MH designed experiments, developed immunostaining methods and analysis procedures, performed image analyses, carried out data analyses, calculated statistics, designed and generated figures and tables, and helped writing the manuscript. JK carried out spatial statistics, generated figures and tables, and helped writing the manuscript. AM designed the experiments, developed immunostaining methods and analysis procedures, designed figures, and wrote the manuscript. I-MK and AW developed immunostaining methods and analysis procedures and helped writing the manuscript. SZ constructed the TMA, performed histopathological examination and p16 expression analysis, and helped writing the manuscript. SG and HS helped writing the manuscript. All authors contributed to the article and approved the submitted version.
