# Supplementary material for: Response to primary chemoradiotherapy of locally advanced oropharyngeal carcinoma is determined by the degree of cytotoxic T cell infiltration within tumor cell aggregates
Source: Front Immunol. 2023 Apr 28;14:1070203. doi: 10.3389/fimmu.2023.1070203 (PMC10175951; doi:10.3389/fimmu.2023.1070203)
Supplement: Supplementary file 1 [file DataSheet_1.docx]

Supplementary Material

**Supplementary Figures**


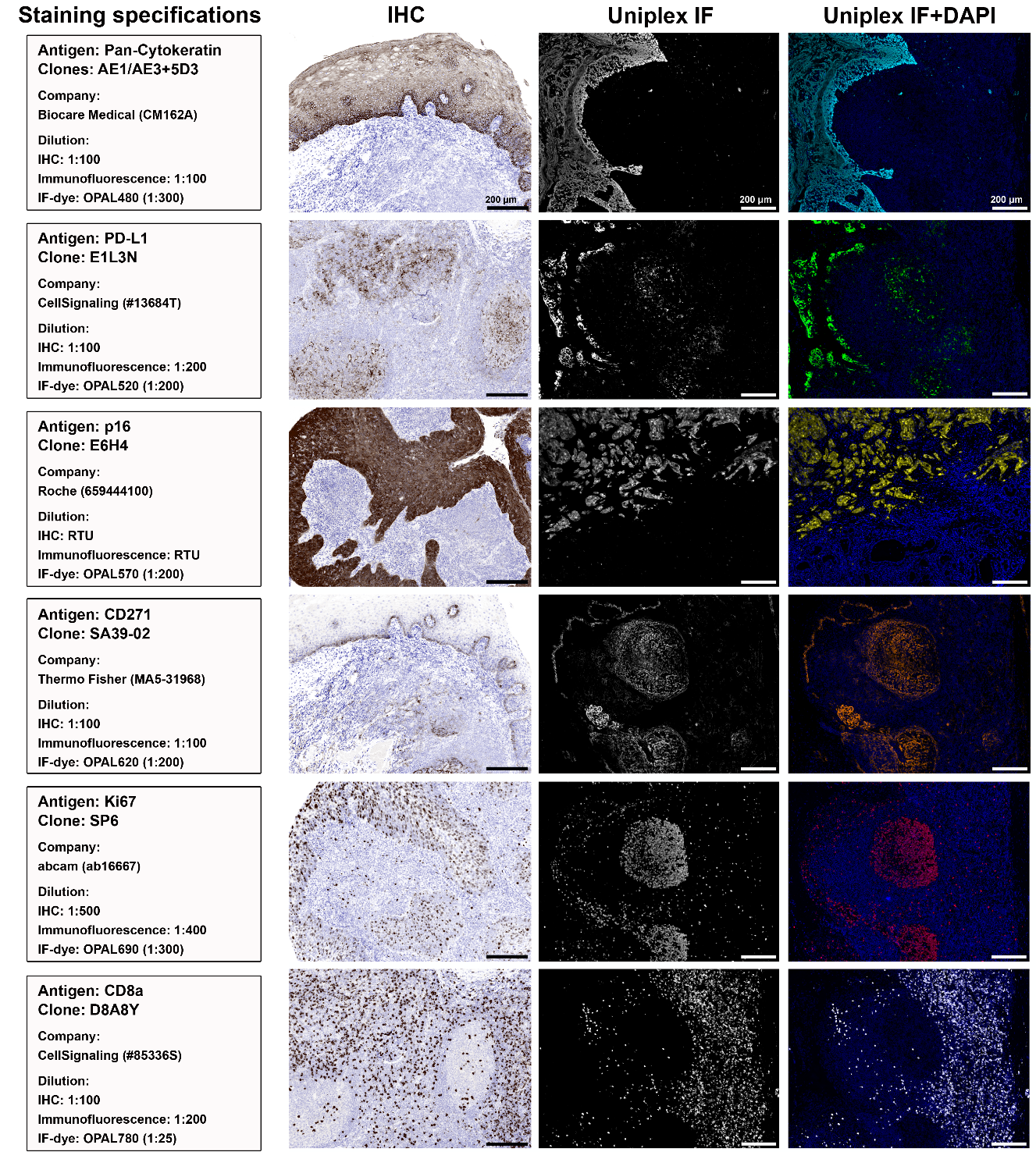


**Supplementary Figure 1: Staining specifications and validation of antibodies used for multiplex immunofluorescence staining.** Antibodies were conjugated to the according OPAL dyes and tested individually along with cross-validation in DAB-IHC (left panel) using the same, non-conjugated antibody clone. To determine the staining position of antibodies and their conjugated OPAL dyes each uniplex IF has been performed three times using various previous heat-retrievals (1x, 3x, 5x) to determine the subsequent position of antibody and conjugated OPAL-dye for multiplex IF. Clones, manufacturers, and staining specifications are listed for each antibody. The center (false grey color fluorescence image) and right panel (uniplex IF with DAPI counterstain) show representative examples of uniplex IF for each individual antibody with at 1x heat-retrieval. Brightness and contrast adjusted. Scale bars in the upper panels apply to all sub-figures. Abbreviations: IHC = immunohistochemistry, IF = immunofluorescence


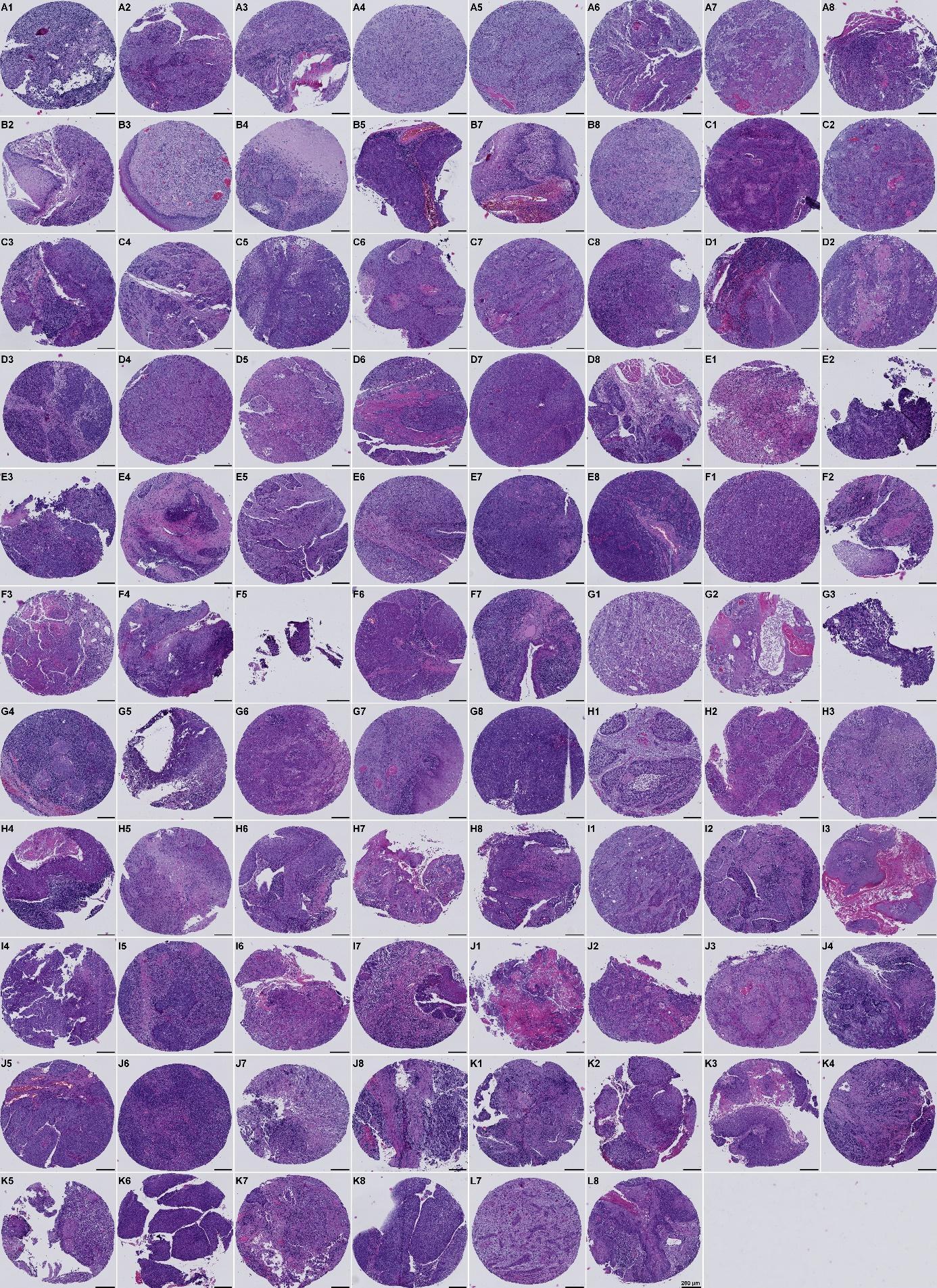


**Supplementary Figure 2: Hematoxylin & Eosin (H&E) stains of the investigated oropharyngeal squamous cell carcinoma TMA.** Cores are arranged as stained on the TMA. Magnification 4.5x. Scale bar in L8 applies to all tumor sub-figures.


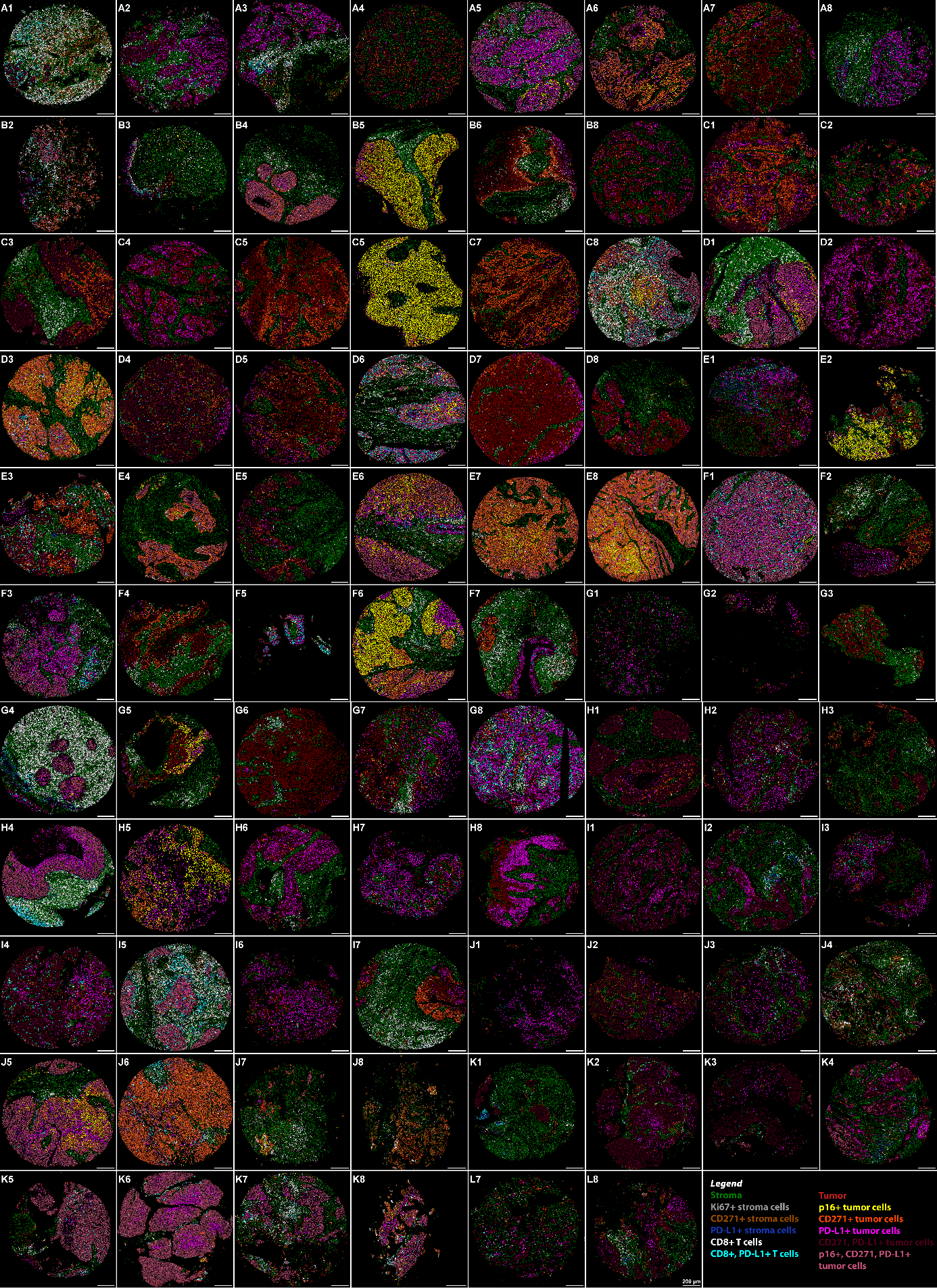


**Supplementary Figure 3: Composite images with an overlay of the color-coded classifier for the identified cell phenotypes for each individual core within the OPSCC TMA.** Cores are arranged as stained on the TMA. Magnification 4.5x. Scale bar in L8 applies to all tumor sub-figures.


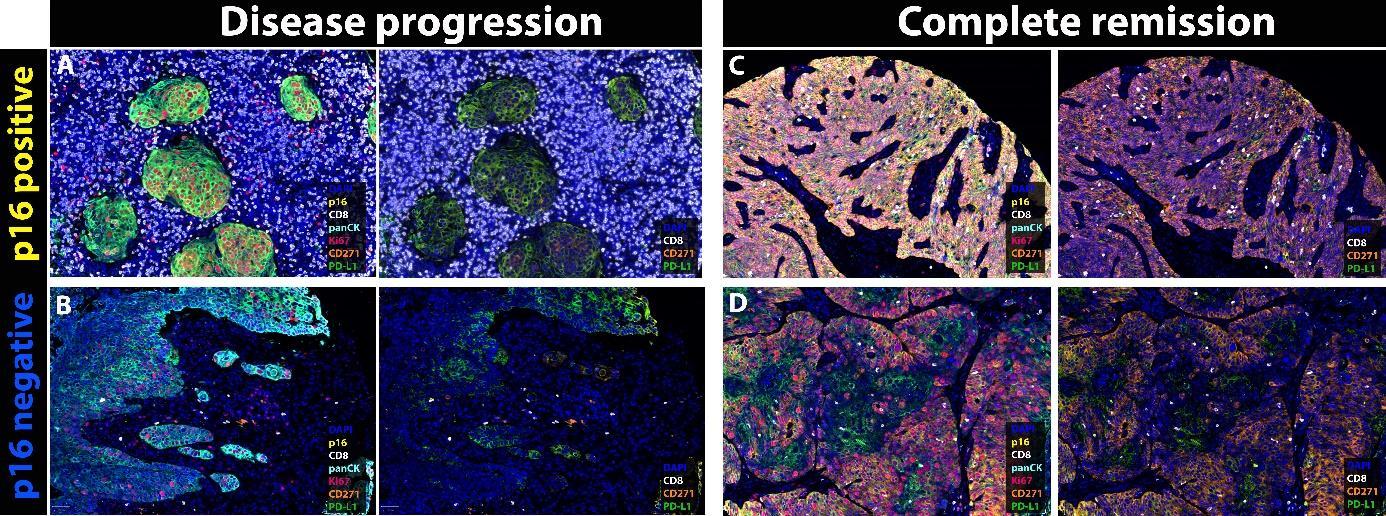


**Supplementary Figure 4: Representative images of four different patients stratified by p16 status with disease progression or complete remission upon chemoradiotherapy**. Despite a strong stromal CD8 infiltrate the patient in (A) experienced a relapse of the primary tumor after only 3 months and died 4 months after initial diagnosis. Notably, CD8 T cells did not infiltrate into the p16 positive tumor which showed almost no CD271 expression. By contrast, (B) shows low levels of CD8 T cell infiltration, CD271 expression and PD-L1 levels. In accordance this p16-negative patient showed a relapse of the tumor 2 months after initiation of RTx and died 4 months after initial diagnosis. In (C) we show the tumor core of a p16 positive patient who is still in survival follow-up 110 months upon initial diagnosis and who did not experience a tumor relapse or progression within the follow-up period. The TME of this patient is characterized by a moderate intratumoral CD8 infiltrate and PD-L1 expression, but a strong CD271 expression within the tumor. Similarly, the p16-negative patient in (D) showed a strong CD271 and PD-L1 expression within the tumor, while the CD8 infiltrate is mainly located within the tumor. This patient is also still in survival follow-up with an overall survival of 139 months. Magnification 6.7x.


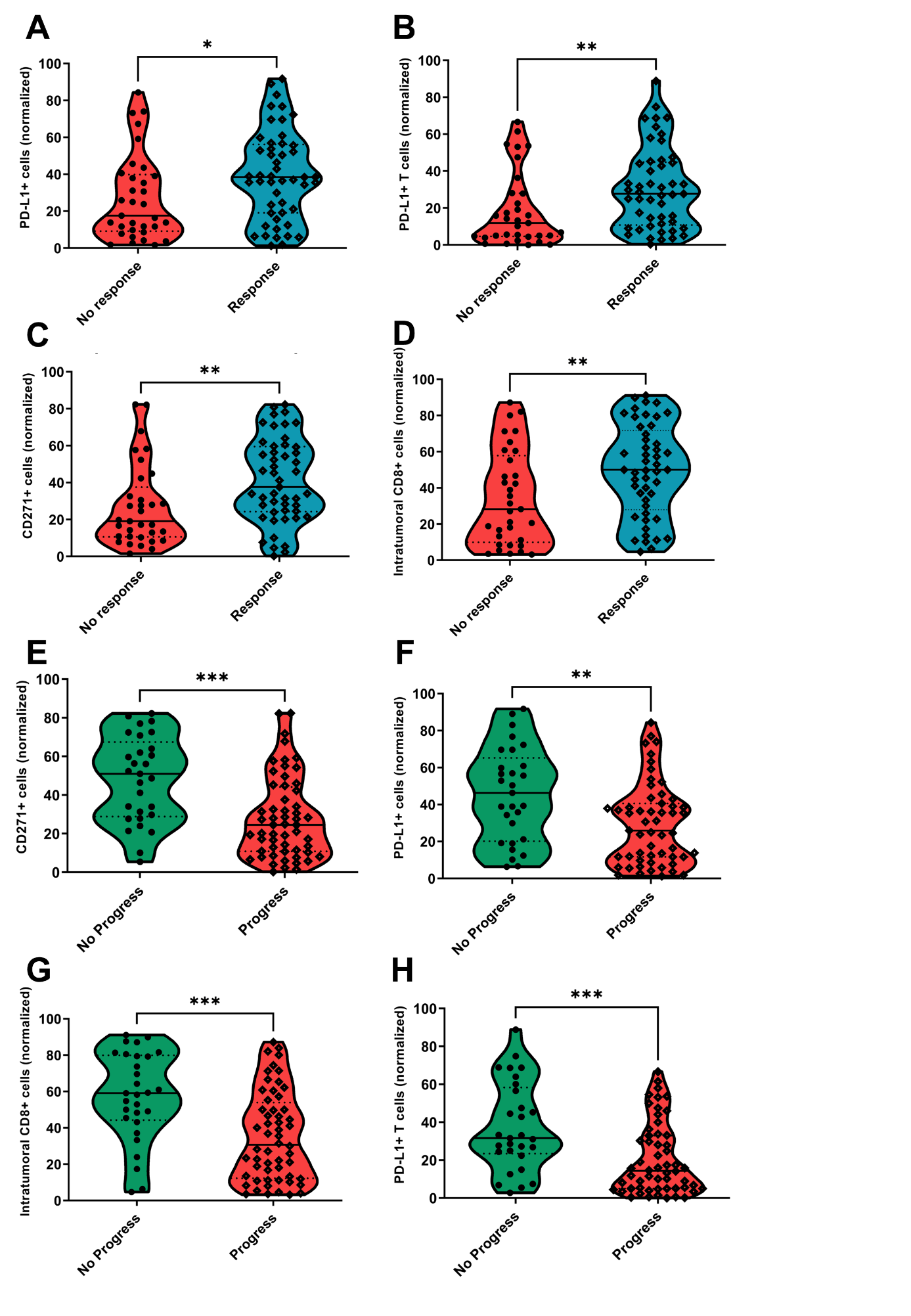


**Supplementary Figure 5: Association between the quantitative biomarker data and the response upon RTx treatment (A-D), as well as their association with tumor relapse during the follow-up period (E-H).** We observed that higher levels of PD-L1 expression where associated with a better response to RCTx and a reduced risk of tumor relapse (A, F). Similarly, a strong intratumoral CD8 T cell infiltration (D, G) and a high PD-L1 expression by CD8 T cells was associated with response to RCTx and a lower risk of tumor relapse (B,H). Abbreviations: * p < 0.05, ** p < 0.005, *** p < 0.001.


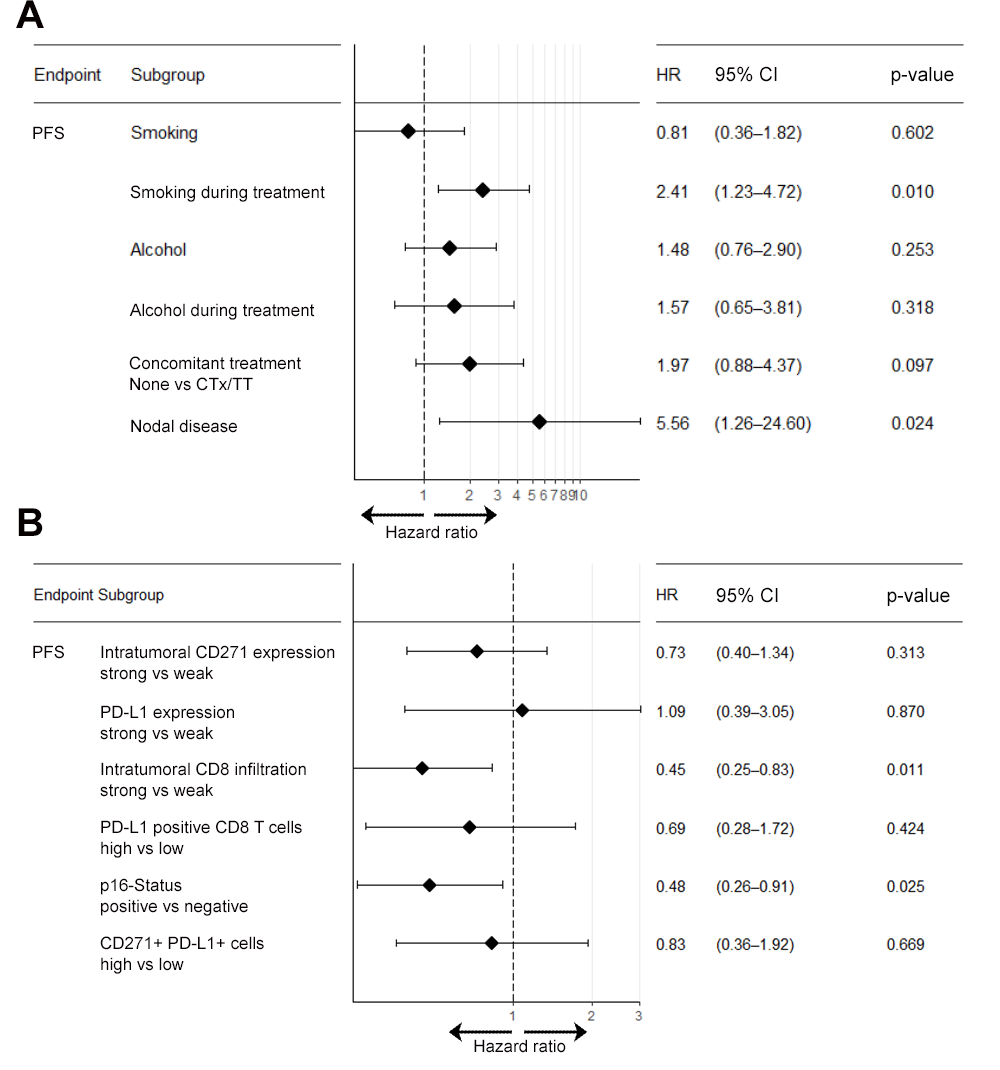


**Supplementary Figure 6:** Results from multivariate Cox-regression analyses for clinical parameters (A) and histopathological parameters (B) found to significantly impact progression-free survival in univariate analysis (A). In this multivariate model, smoking during treatment and nodal disease were again significantly associated with a shorter progression-free survival (PFS) whereas among a higher infiltration by intratumoral CD8 T cells was significantly associated with PFS. Abbreviations: CI = 95% confidence interval; HR = hazard ratio; PFS = progression-free survival


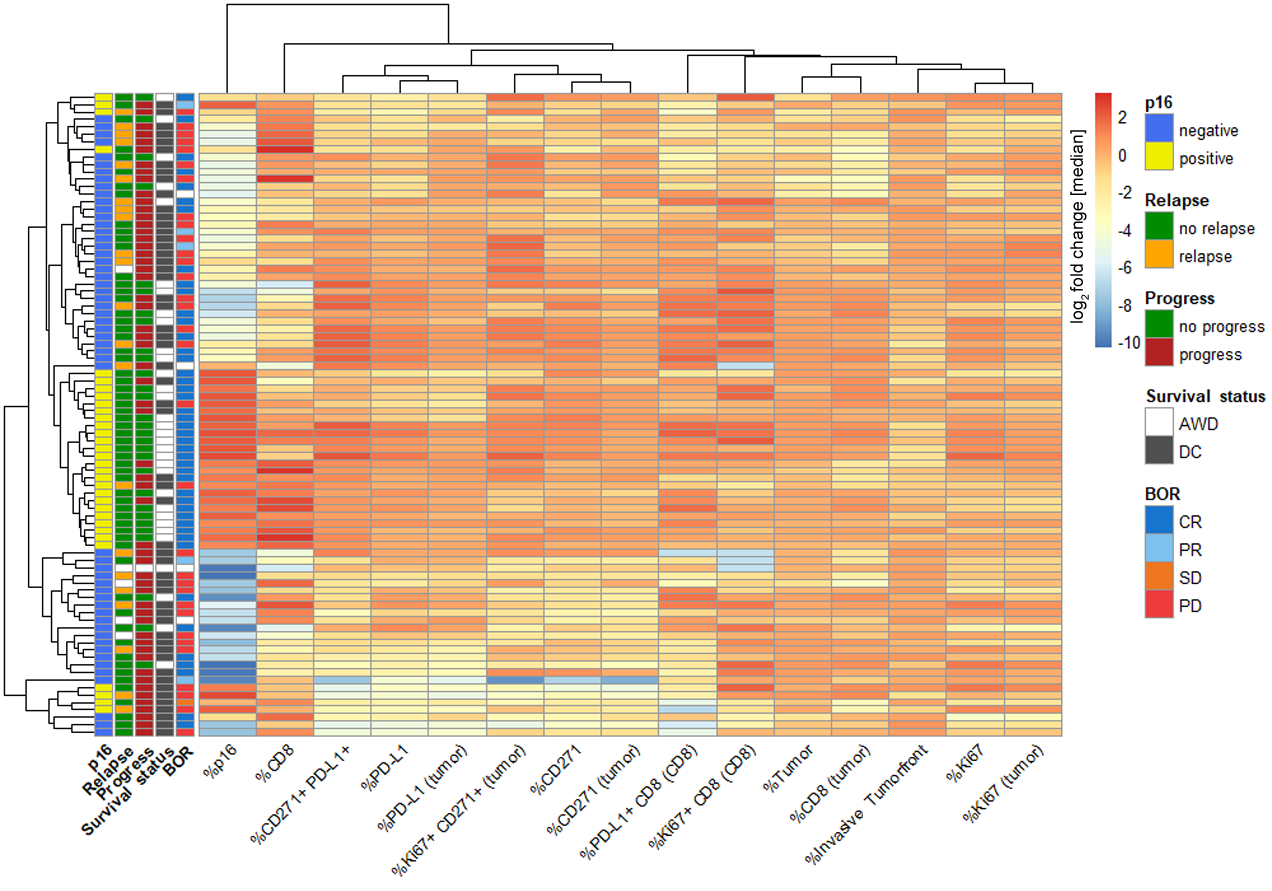


**Supplementary Figure 7: Heatmap depicting the association between the individual patients´ p16-status, best overall response (BOR) to RCTx, survival status with the expression of the investigated biomarkers in the according OPSCC tumor samples.** Expression of the investigated biomarkers in indicated as log(2) fold change of the median marker expression in each tumor sample. It can be found that p16 positive tumors overall show a stronger CD8 infiltration, a smaller amount of invasive tumor front and a higher infiltration by PD-L1 positive CD8 T cells. Abbreviations: AWD = alive with disease; BOR = best overall response; CR= complete response; DC = deceased; PD = progressive disease; PR = partial response


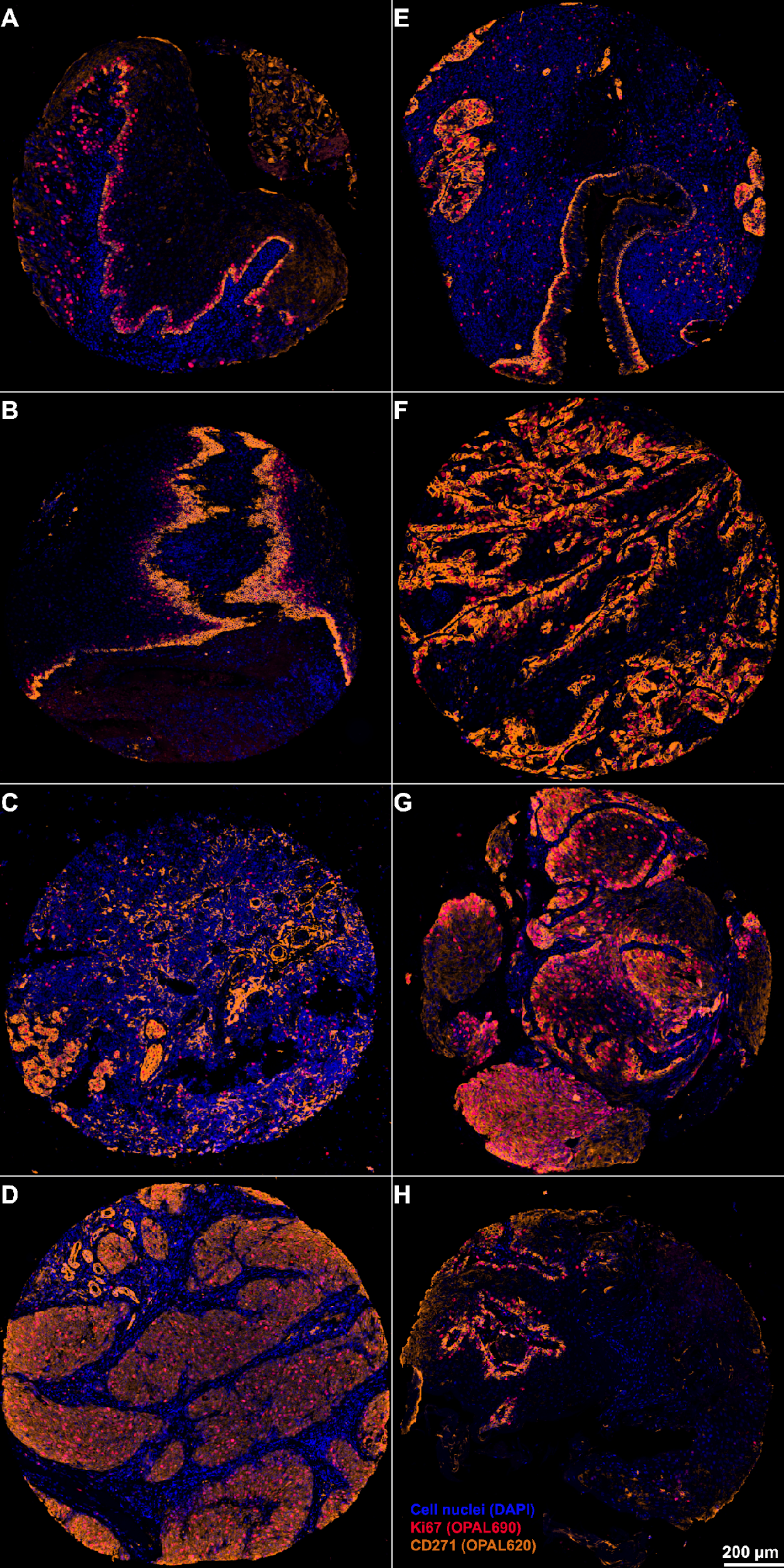


**Supplementary Figure 8: Representative examples from 8 different patients for the expression pattern of the stem cell marker CD271 and the proliferation marker Ki67 in the OPSCC tumor microenvironment.** The expression of CD271 is mainly confined to the tumor compartment (A-H) and a substantial co-localization with Ki67 can be observed for most tumor tissues. In most samples CD271 also shows an expression pattern that clearly reflects the stem cell properties of tumor cells (A, B, E – H). Expression in stroma cells is sometimes present as well, reflecting the stem cell-like properties of endothelial-mesenchymal cells (C, D). In a minority of tumor samples there is a rather homogenous weak or moderate expression of CD271 within the tumor compartment (D). Magnification 3.6x. Scale bar in H applies to all panels of the figure.

**
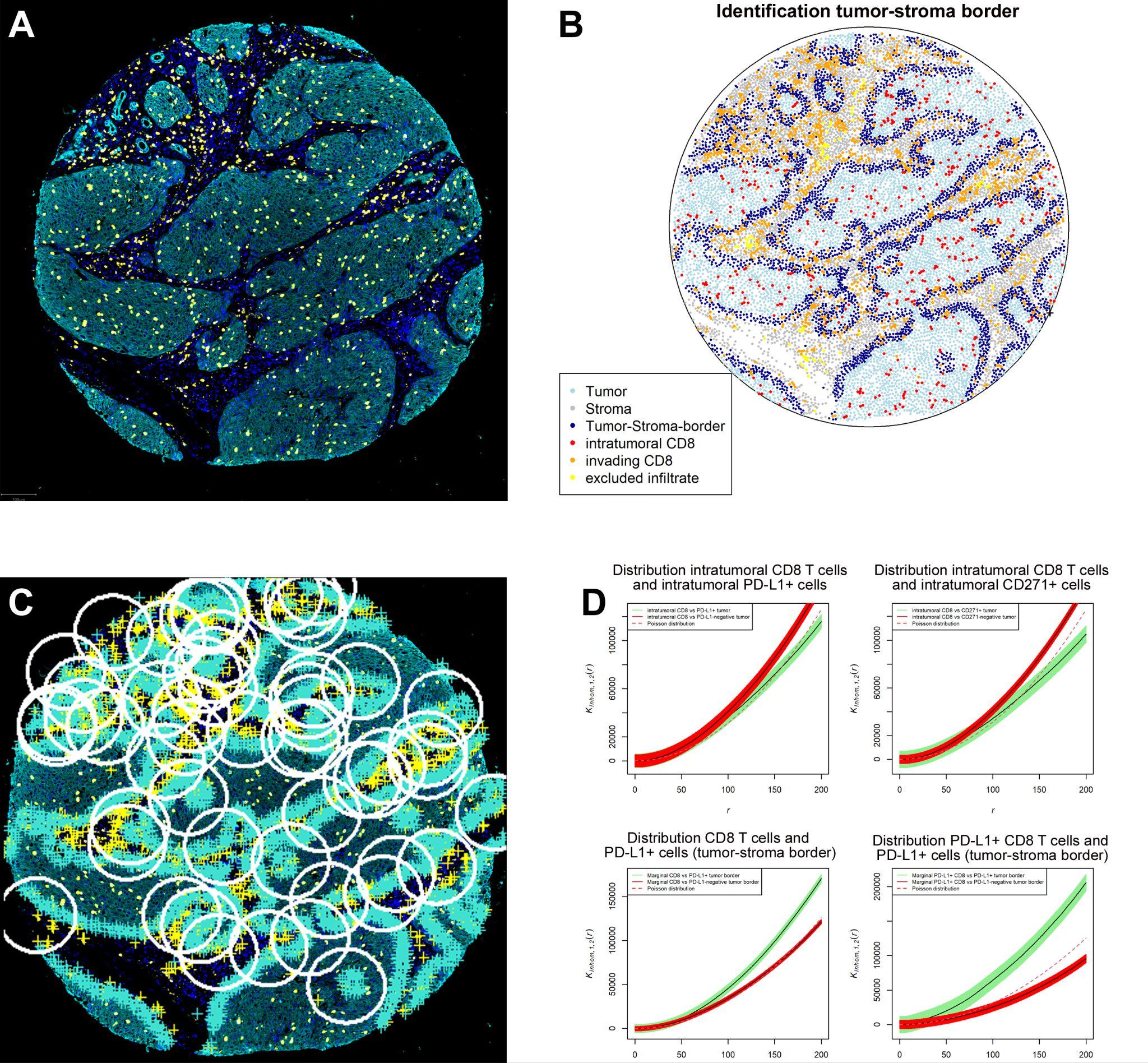
**

**Supplementary Figure 9: Representative procedure to identify the tumor-stroma border region, the distribution of CD8 T cells therein, and their spatial interaction with PD-L1 and CD271 positive cells.** (A) depicts the multiplex image of a p16 positive patient with pan-cytokeratin (crystal blue), CD8 T cells (yellow) and cell nuclei (blue, DAPI). Identification of the tumor-stroma border was based on a nearest neighbor-approach defining every tumor cell that is located within 35 µm of a stroma cell as a tumor cell within the invasive tumor front (B). Subsequently CD8 T cells have been reclassified according to their spatial localization within the tumor (intratumoral), tumor-stroma border region (invading) or the stroma itself (excluded). (C) For the Kcross-function, the average number of neighboring cell events within a circle of a given radius r is calculated for each cell event in the population. Here, representative circles of r = 100 µm are shown around a random selection of CD8-CTL (yellow crosses). This process is repeated for every cell for every value of r up to 200 µm in the region of interest (ROI) and cell events of the other population (in this case PD-L1+ cells of the invasive tumor front; cyan crosses) within the circles are counted. K-functions are standardized, and corrections for edge effects are performed, the details of which have been explained before. (D) Comparison between Kcross-functions used for the analysis of different hypotheses for marker positive (green) and negative (red) cells. Divergence of the curves at any point suggests a significant interaction between different cell populations. Interpretation of the K-function has been explained in detail before (1).

**
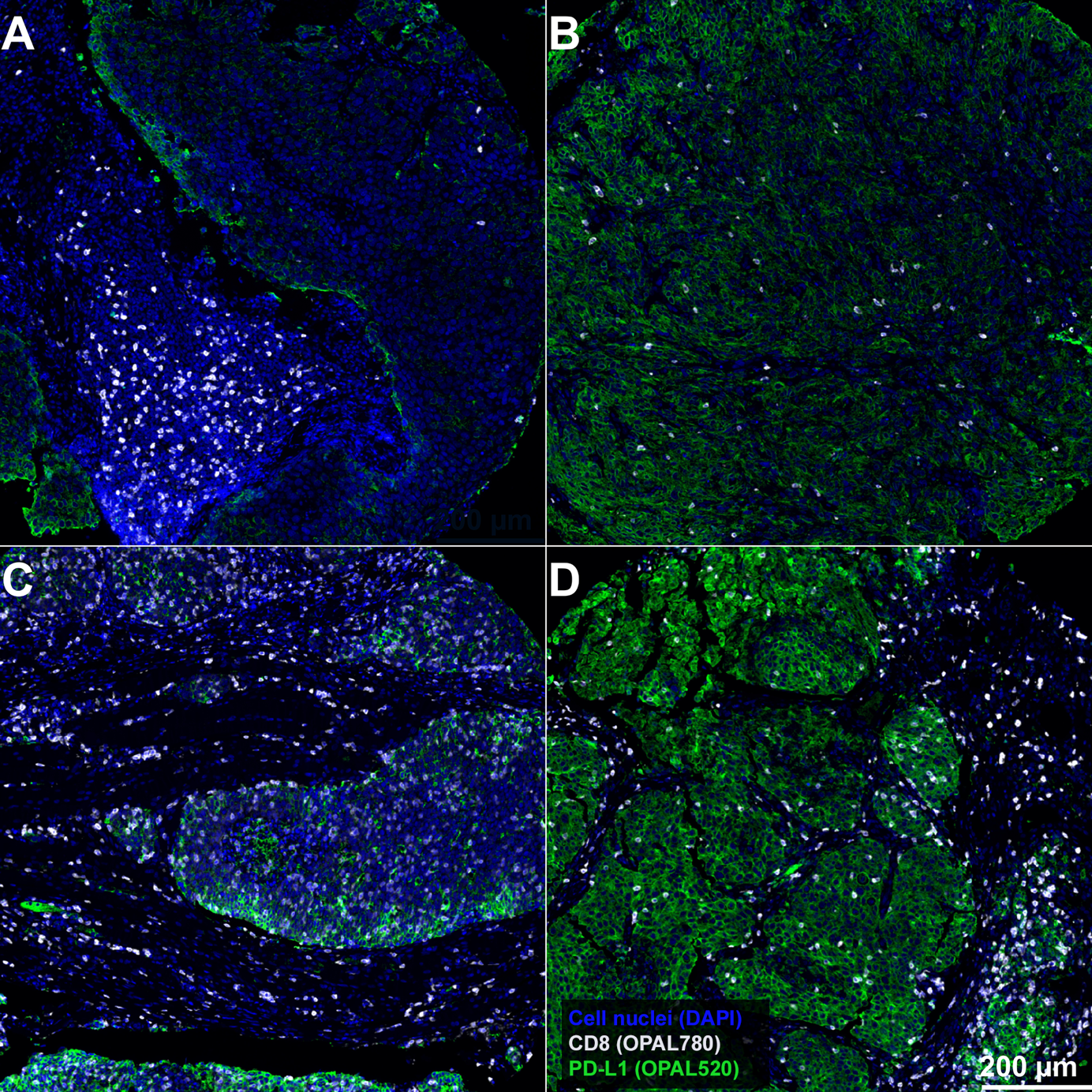
**

**Supplementary Figure 10: Representative images of PD-L1 expression within the TME of the investigated OPC cohort stratified by PD-L1 expression at the tumor periphery (A, C) and a diffuse PD-L1 staining pattern across the entire tumor cell compartment.** The majority of tumor samples presented with a PD-L1 expression predominantly confined to the tumor periphery, whereas approx. 30% of patient samples showed a strong expression within the entire tumor cell compartment, termed “diffuse” staining pattern. With regard to CTL infiltration, we did not observe significant differences between both staining patterns. Also, we did not find significant differences for the investigated staining patterns between HPV-positive and HPV-negative OPC patients. Magnification 5x. Scale bar in D applies to all panels of the figure.


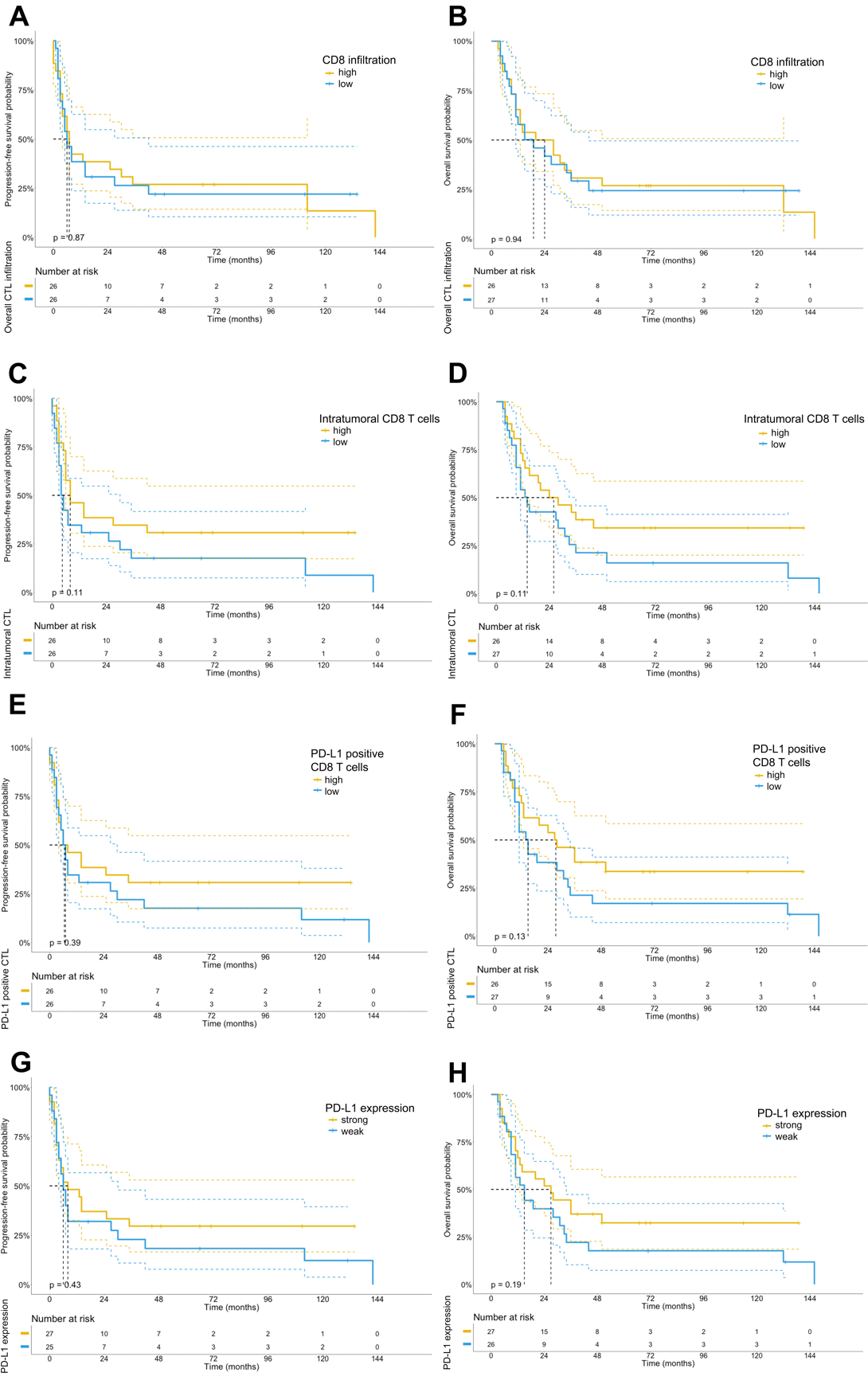


**Supplementary Figure 11: Kaplan Meier survival plots for p16-negative patients within the investigated OPSCC cohort stratified by the median of the biomarker expression levels.** In accordance with our previous observations in the overall patient cohort, overall CD8 T cell numbers were not significantly associated with better survival outcomes (median OS: 15.0 vs 28.0 months, p=0.94) **(A, B).** Although the number of tumor-infiltrating CD8 T cells stratified by the median (median OS: 14 vs 26.0 months, p=0.11) showed a trend towards a better OS, this was statistically below significance. Furthermore, overall PD-L1 expression levels (median OS: 15.0 vs 27.0 months, p=0.19; **G, H**) and the number of PD-L1 positive CD8 T cells (median OS: 15.0 vs 27.5 months, p=0.13; **E, F**) were not significantly associated with favorable survival outcomes as previously found in the overall patient cohort.

**Supplementary Tables**

**Supplementary Table 1:** Definition of real-world endpoints applied in this study.

| **Endpoint** | **Outcome** |
| --- | --- |
| **Primary** |  |
| Overall survival (OS) | The time interval from index date to date of death.  Patients alive at the date of last contact were censored.  Index dates were defined as initial diagnosis of OPSCC |
| **Secondary** |  |
| Best-overall response (BOR) | Best tumor response defined as complete response (CR), partial response (PR), stable disease (SD), or progressive disease (PD) based on real-world response assessments* |
| Progression-free survival (PFS) | The time interval from start of radiation therapy to physician-reported date of progression, relapse, death date or start date of a new treatment due to progression of disease (whichever event occurred first). Patients without a relapse, progression event or date of death were censored at the date of last contact. |
| Recurrence of primary tumor | Relapse of the tumor at the primary tumor site after start of primary radiotherapy treatment |
| Tumor progression | Any of the following events has been defined as tumor progression: relapse, locoregional or metastatic progress of the tumor after start of primary radiotherapy |

* Complete response: complete resolution of all visible disease; partial response: disease still present, with partial reduction in size of visible disease in some or all areas without any areas of increase in visible disease; stable disease: no change in overall size of visible disease or mixed response.

**Supplementary Table 2:** Key Resources and Materials.

| **Reagent or Resource** | **Manufacturer** | **Identifier** |
| --- | --- | --- |
| **Chemicals and Reagents** | | |
| Xylol (Isomere) | Carl-Roth GmbH +Co. KG (in the following termed Carl-Roth GmbH) | 9723.5 |
| 99,8 % EtOH | Carl Roth GmbH | K928.3 |
| 96 % EtOH | Carl Roth GmbH | T171.4 |
| 70 % EtOH (dilution from 99,8 % ETOH) | Carl Roth GmbH | - |
| Tris-HCl (Pufferan) p.a. | Carl Roth GmbH | 9090.3 |
| NaCl (99,8%) | Carl Roth GmbH | 9265.1 |
| Formalin solution (neutral buffered 10 %) | Sigma-Aldrich | HT501128-4L |
| Fluorescence Mounting Medium | Agilent DAKO | S302380-2 |
| OPAL Polaris 7-Color (manual Kit) | Akoya Biosciences | NEL861001KT |
| Opal Polymer HRP (Ms+Rb) | Akoya Biosciences | ARH1001EA |
| Opal 480 | Akoya Biosciences | - |
| Opal 520 | Akoya Biosciences | - |
| Opal 570 | Akoya Biosciences | - |
| Opal 690 | Akoya Biosciences | - |
| Opal 620 | Akoya Biosciences | - |
| Opal 780 | Akoya Biosciences | - |
| TSA-Dig | Akoya Biosciences | - |
| DAPI | Akoya Biosciences | FP1490 |
| Antibody Diluent | Akoya Biosciences | ARD1001EA |
| Amplification Diluent | Akoya Biosciences | IF1498 |
| AR9 Buffer | Akoya Biosciences | AR900250ML |
| ImmEdge_Pen (hydrophobic barrier pen) | Vector Laboratories | H-4000 |
| Aqua_rinsing solution (Ampuwa) | Fresenius Kabi Deutschland | 1088811 |
| AVS Titrinorm pH 4 | Avantor delivered by VWR | 32095.264 |
| AVS Titrinorm pH 7 | Avantor delivered by VWR | 32096.267 |
| AVS Titrinorm pH 9 | Avantor delivered by VWR | 32039.261 |
| EDTA >99 % | Carl Roth GmbH | CN06.2 |
| Tris Pufferan >99 % p.a. | Carl Roth GmbH | 4855.2 |
| Eukitt (mounting medium) | Avantor delivered by VWR | 03989-100 ml |
| PBS (without Ca^2+^, Mg^2+^) | Bio&Sell | BS.L.182-50 |
| tri-Natriumcitrate (Dihydrat) | Carl Roth GmbH | 3580.1 |
| Hydrogen peroxide 3 % | neoLab | LC-10300.1 |
| Citric acide (Monohydrate) | Carl Roth GmbH | 5110.1 |
| Hematoxylin | Agilent (DAKO) | CS700 |
| ImmPRESS HRP Horse Anti-Mouse IgG (Kit) | Vector Laboratories | MP-7402 |
| ImmPRESS HRP Horse Anti-Rabbit IgG (Kit) | Vector Laboratories | MP-7401 |
| ImmPACT_DAB Substrate, HRP | Vector Laboratories | SK-4105 |
| Dulbecco`s PBS w/o Ca,Mg | Sigma-Aldrich | D8537-500 ml |
|  |  |  |
| **Antibodies and Proteins** | | |
| Pan Cytokeratin Plus [AE1/AE3+5D3] | Biocare Medical | CM162A |
| p16 INKa [E6H4] | CINtec (Roche) | 659444100 |
| Ki67 [SP6] | abcam | ab16667 |
| CD8a [D8A8Y] | Cell Signaling | 85336S |
| CD271 [SA39-02] | Thermo Fisher Scientific | MA5-31968 |
| PD-L1 [E1L3N] | Cell Signaling | 13684T |
| **Critical Commercial Instruments, Consumables, Kits and Assays** | | |
| Vectra Polaris Imaging System | Akoya Biosciences | CLS143455 |
| Nanozoomer 2.0HT | Hamamatsu Photonics | C9600 |
| Thermomixer Compact | Eppendorf | 535025873 |
| pH-meter (Lab 850) | Schott Instruments | 6110160 |
| Balance (Adventurer Pro AV2101) | Ohaus | 8727143362 |
| Fine balance (Extend) | Sartorius | ED124S-OCE |
| Stirrer (MR3000) | Heidolph | 119907925 |
| Microwave oven | Bosch | HMT75M451 |
| Pipet_(Eppendorf research 1000 µl) | Eppendorf | 4855156 |
| Pipet_(Eppendorf research 100 µl | Eppendorf | 1264836 |
| Pipet_(Eppendorf research 10 µl | Eppendorf | 4117065 |
| Stain Tray black | Carl-Roth GmbH | HA51.1 |
| Timer | Macherey-Nagel | 140277-006 |
| Heating oven (TV-40u) | Memmert (Schwabach) | 770633 |
| Freezer | Liebherr | 7083245-00 |
| Shaker (Polymax 1040) | Heidolph | 543-42205-00 |
| Färbekästen aus Kalk  Soda Glas | Carl-Roth GmbH | H554.1 |
| Dye box for 10 slides | Carl-Roth GmbH | H552.1 |
| Dye box and inserts (PMP) | Carl-Roth GmbH | 2290.2 / 2291.2 |
| Pipet tips (10/20 µl XL graduated tip) | Starlab Group | S1110-3700 |
| Pipet tips (1000 µl blue graduated tip | Starlab Group | S1111-6701 |
| Pipet tips (200 µl yellow tip) | Starlab Group | S1111-0706 |
| Lab gloves Nitra Touch | Ansell | 4400053 |
| U-gloves Nitril Blue | Abena Nova | 290419 |
| Cover glass tweezer 18/8 | Carl-Roth GmbH | K718.1 |
| Powder funnel | Vitlab | 71094 |
| Rotilabo  Weighing bowls (89*89mm) | Carl-Roth GmbH | 2150.1 |
| Alufoil | Carl-Roth GmbH | AAI 76.1 |
| Lab bottle (1000 ml) | Schott Duran | 21820545 |
| Stirring rods | Carl-Roth GmbH | PK77.1 |
| Kimtech science | Kimberly-Clark | 7552 |
| 3M KCl | SI Analytics | - |
| 4 N NaOH | Carl-Roth GmbH | T198.1 |
| 1 M HCl | Carl-Roth GmbH | K025.1 |
| Cellstar Tubes (15ml) | Greiner Bio-one | 188271 |
| Reaction tubes 1,5ml | Greiner Bio-one | 618201 |
| Microscope slides ThermoScientific (Superfrost Plus) | Menzel GmbH | 1800AMNZ |
| **Biological Samples** | | |
| Tissue microarray | Institute of Pathology, University Medical Center Mainz | N/A |
| FFPE tissue blocks | Institute of Pathology, University Medical Center Mainz | N/A |
| **Deposited Data** | | |
| Quantitative data table | Uploaded on Dryad: <https://doi.org/10.5061/dryad.95x69p8p8> |  |
| Single-cell coordinates table | Uploaded on Dryad: <https://doi.org/10.5061/dryad.95x69p8p8> |  |
| **Software and Algorithms** | | |
| ImageJ (Fiji version 2.0.0) | <https://imagej.net/> | N/A |
| QuPath version 0.3.2. | <https://qupath.github.io/> | (2) |
| R version 4.0.3 | <https://cran.r-project.org/bin/windows/base/> | N/A |
| R studio desktop, version 1.1.423 | <https://www.rstudio.com/> | N/A |
| Spatial analysis | [www.spatstat.org](http://www.spatstat.org) | (3) |
| Survival R package | <https://cran.r-project.org/web/packages/survival/index.html> | (4) |
| Pheatmap package | <https://cran.r-project.org/web/packages/pheatmap/index.html> | N/A |
| The Human Protein Atlas | <http://www.proteinatlas.org/> | (5) |

**Supplementary Table 3.** Multiplex IF protocol including staining sequence, applied buffers, antibodies and fluorophores as well as resulting staining patterns.

| No | Antigen retrieval buffer | Antigen (clone) | Primary AB  (species, dilution, incubation) | Fluorochrome (Cat.-No.) | Fluorochrome  (dilution, incubation) | Staining pattern |
| --- | --- | --- | --- | --- | --- | --- |
| 1 | AR2 (Tris-EDTA/pH9) | Ki67 [SP6] | rabbit (mono), 1:400, for 1 h at 28 - 30°C | OPAL690 | 1:300, for 10min at 24 - 26°C | Nuclear |
| 2 | AR2 (Tris-EDTA/pH9) | Pan-Cytokeratin  [AE1/AE3 +5D3] | mouse (mono), 1:100, overnight at 4°C | OPAL480 | 1:200, for 10min at 24 - 26°C | Membranous/ cytoplasm |
| 3 | AR2 (Tris-EDTA/pH9) | CD271  [SA39-02] | Rabbit (mono), 1:100, for 1 h at 28 - 30°C | OPAL620 | 1:300, for 10min at 24 - 26°C | Membranous |
| 4 | AR2 (Tris-EDTA/pH9) | PD-L1 [E1L3N] | rabbit (mono), 1:200, overnight at 4°C | OPAL520 | 1:200, for 10min at 24 - 26°C | Membranous |
| 5 | AR2 (Tris-EDTA/pH9) | p16 [E6H4] | mouse  (mono), RTU, for 1 h at 28 - 30°C | OPAL570 | 1:200, for 10min at 24 - 26°C | Nuclear / cytoplasm |
| 6 | AR2 (Tris-EDTA/pH9) | CD8 [D8A8Y] | rabbit (mono), 1:200, overnight at 4°C | TSA-Dig and OPAL780 | 1:100 for 10min at 24 - 26°C and 1:25 for 60min at 24 - 26°C | Membranous |
| 7 | N/A | DAPI | for 5min at 24-26°C | N/A | N/A | Nuclear |

**Supplementary Table 4.** Markers used for detection and quantification of cell phenotypes within the OPSCC tissue microarray.

| **Cell phenotype** | **Markers for detection/quantification** |
| --- | --- |
| Tumor cells | Pan-Cytokeratin (+) |
| HPV positive tumor cells | Pan-Cytokeratin (+) p16 (+) |
| Tumor stem cells | Pan-Cytokeratin (+) CD271 (+) |
| Proliferative tumor cells | Pan-Cytokeratin (+) Ki67 (+) |
| Proliferative tumor stem cells | Pan-Cytokeratin (+) Ki67 (+) CD271 (+) |
| Immune evasive tumor cells | Pan-Cytokeratin (+) PD-L1 (+) |
| Immune evasive tumor stem cells | Pan-Cytokeratin (+) CD271 (+) PD-L1 (+) |
| Cytotoxic T cells (CTL) | CD8 (+) |
| Proliferating CTL | CD8 (+) Ki67 (+) |
| Exhausted CTL | CD8 (+) PD-L1 (+) |

**Supplementary Table 5:** Comparison of quantitative data dichotomized by p16 status of OPSCC samples.

| Quantitative Parameters | Overall patient cohort | p16 positive OPSCC patients | p16 negative OPSCC patients | p-value |
| --- | --- | --- | --- | --- |
| Tumor volume (%) | 60.3 | 65.8 | 58.7 | 0.463 |
| CD8 infiltrate (%) | 5.1 | 9.4 | 2.8 | **<0.001** |
| Ki67 expression (%) | 19.6 | 19.4 | 20.0 | 0.406 |
| CD271 expression (%) | 29.2 | 37.6 | 24.6 | **0.026** |
| PD-L1 expression (%) | 35.5 | 35.7 | 35.4 | 0.737 |
| Intratumoral CTL | 43.1 | 49.1 | 40.0 | 0.156 |
| Proliferative tumor | 31.8 | 33.0 | 30.1 | 0.480 |
| Proliferative CTL | 11.3 | 10.9 | 11.7 | 0.872 |
| Tumor stem cells | 63.2 | 69.0 | 44.6 | 0.083 |
| Stroma stem cells | 2.5 | 2.5 | 2.5 | 0.993 |
| Immune evasive tumor cells | 64.4 | 54.8 | 65.2 | 0.530 |
| Exhausted CTL | 22.4 | 24.4 | 20.0 | 1.0 |
| Proliferative exhausted CTL | 8.0 | 8.0 | 6.9 | 0.703 |
| Evasive stem cells | 17.6 | 21.7 | 15.3 | 0.442 |

**Supplementary Table 6:** Results from non-parametric correlation analysis using spearman-rho testing.

| **Investigated parameters** | **Correlation coefficient** | ***p*-value** |
| --- | --- | --- |
| CD8 vs p16 expression | 0.358 | **<0.001** |
| Ki67 vs p16 expression | 0.226 | **0.037** |
| CD271 vs p16 expression | 0.414 | **<0.001** |
| CD271 vs Ki67 expression | 0.330 | **0.002** |
| PD-L1 vs p16 expression | 0.147 | 0.178 |
| CD8 vs PD-L1 | -0.055 | 0.616 |
| Ki67 vs PD-L1 | 0.309 | **0.004** |
| CD8 vs Ki67 | -0.211 | 0.051 |
| Intratumoral CD8 vs Ki67 | 0.351 | **<0.001** |
| Intratumoral CD8 vs intratumoral Ki67 | -0.390 | **<0.001** |

**Supplementary Table 7:** Univariable Cox-regression analysis for progression-free survival stratified by clinical-pathological parameters and biomarker variables.

| **Parameters** | **Subgroups** | **HR** | **95%CI** | **p-value** |
| --- | --- | --- | --- | --- |
| *A Clinical-pathological parameters* | | | | |
| Age (years) | >61 vs ≤61 | 0.83 | 0.49-1.42 | 0.51 |
| Gender | Male vs female | 1.08 | 0.61-1.91 | 0.77 |
| Grading | ≤G2 vs >G2 | 0.61 | 0.34-1.11 | 0.104 |
| Smoking | Yes vs no | 2.02 | 1.06-3.85 | **0.032** |
| Smoking during treatment | Yes vs no | 3.19 | 1.83-5.58 | **<0.001** |
| Smoking quantity | >30PY vs ≤30 PY | 2.12 | 1.18-3.82 | **0.015** |
| Alcohol | Yes vs no | 2.00 | 1.14-3.53 | **0.017** |
| Alcohol during treatment | Yes vs no | 2.71 | 1.3-5.66 | **0.008** |
| Alcohol quantity | Heavy vs moderate | 2.64 | 1.38-5.05 | **0.004** |
| T-stage | >T2 vs ≤T2 | 1.19 | 0.56-2.53 | 0.64 |
| N-stage | >N1 vs ≤N1 | 3.27 | 1.18-9.05 | **0.023** |
| Neck dissection | Yes vs no | 0.91 | 0.51-1.62 | 0.76 |
| Concomitant treatment | No treatment vs concomitant treatment with CTx or TT | 1.97 | 1.03-3.76 | **0.040** |
| BOR to RTx | Response vs no response | 0.09 | 0.04-0.176 | **<0.001** |
| *B Biomarker parameters* | | | | |
| p16-status^1^ | Positive vs negative | 0.37 | 0.20-0.68 | **0.0013** |
| Tumor volume^2^ | High vs low | 0.50 | 0.29-0.86 | **0.012** |
| CTL infiltration^2^ | Strong vs weak | 0.75 | 0.44-1.29 | 0.30 |
| CD271 expression^2^ | Strong vs weak | 0.40 | 0.23-0.70 | **0.001** |
| PD-L1 expression^2^ | Strong vs weak | 0.59 | 0.34-0.99 | **0.049** |
| Ki67 expression^2^ | Strong vs weak | 0.80 | 0.46-1.36 | 0.41 |
| Intratumoral CTL^2^ | High vs low | 0.35 | 0.20-0.60 | **<0.001** |
| Ki67+ tumor cells^2^ | High vs low | 0.78 | 0.45-1.32 | 0.36 |
| PD-L1 tumor cells^2^ | High vs low | 1.02 | 0.60-1.72 | 0.95 |
| CD271+ tumor cells^2^ | High vs low | 0.59 | 0.34-1.0 | 0.052 |
| CD271+ stroma cells^2^ | High vs low | 0.49 | 0.29-0.84 | **0.01** |
| PD-L1+ CTL^2^ | High vs low | 0.44 | 0.26-0.76 | **0.003** |
| CD271, PD-L1 positive cells | High vs low | 0.44 | 0.26-0.76 | **0.003** |

^1^ = dichotomization was performed according to positivity or negativity of p16 staining in IHC; ^2^ groups were separated according to the median percentage of marker-feature positive cells within the patient cohort. The relative abundance of cell types that were characterized by more than a single variable (i.e., intratumoral CTL) was referred to the parent cell population (i.e., all CTL for intratumoral CTL or all tumor cells in case of Ki67 positive tumor cells). The p value is indicated in bold numbers when statistically significant. Abbreviations: BOR = best overall response, HR = hazard ratio, CI =confidence interval, CTL = cytotoxic T lymphocytes

**Supplementary Table 8:** Results from log-rank test comparing the median overall survival and progression-free survival subsequent to dichotomization of the patient cohort stratified by biomarker expression. Chi-square tests was used to test the association between the biomarker groups and the best-overall response (RemiAll) to RTx and the event of disease progression.

| Biomarker | Median OS | *p*-value | Median PFS | *p*-value | *p*-value BOR | *p*-value Progress |
| --- | --- | --- | --- | --- | --- | --- |
| Tumor volume | 20 months (4.5-35.5) vs 88 months (15.7-160.3) | **0.021** | 7 months (1.7-12.3) vs 32.8 (0-106.2) | **0.010** | 0.093 | **0.022** |
| CTL | 25 months (7.9 vs 42.1) vs 50 months (0-108.1) | 0.271 | 8 months (0-16.2) vs 33 months (0-95.1) | 0.30 | 0.745 | 0.649 |
| p16 status | 20.0 months (5.1-34.9) vs 136.0 months (22.6-249.4) | **0.001** | 6.0 months (1.2-8.4) vs 134.0 months (21.2-246.8) | **<0.001** | **0.012** | **0.002** |
| p16 expression | 19 months (4.5-33.5) vs 136 months (50.1-221.9) | **<0.001** | 6 months (3.5-8.5) vs 134 (21.1-246.9) | **<0.001** | **0.009** | **0.003** |
| Ki67 | 31 months (19.97-42.0) vs 39 months (N/A) | 0.287 | 9 months (0-26.9) vs 14 months (0-42.9) | 0.402 | 0.599 | 0.171 |
| CD271 | 18 months (6.6-29.4) vs 132 months (15.7-248.3) | **0.001** | 6 months (3.2-8.8) vs 112 months (16.1-207.9) | **<0.001** | **0.002** | **0.001** |
| PD-L1 | 19 months (3.5-34.5) vs 88 months (6.9-169.1) | **0.018** | 7 months (3.8-10.2) vs 40 months (0-109.6) | **0.048** | **0.017** | 0.067 |
| Tumor-infiltrating CTL | 15 months (7.6-22.4) vs NR | **<0.001** | 5 months (1.8-8.2) vs 85 months (NA) | **<0.001** | **<0.001** | **<0.001** |
| Ki67+ tumor cells | 33 months (22.3-42.7) vs 36 months (0-89) | 0.449 | 9 months (0-26.9) vs 14 months (0-47.4) | 0.349 | 0.788 | 0.171 |
| Proliferating CD8 CTL | 28 months (8.4-47.6) vs 41 months (NR) | 0.126 | 9 months (0-32.9) vs 14 months (0-53.0) | 0.212 | 0.323 | 0.067 |
| CD271+ tumor cells | 28 months (13.3-42.7) vs 132 months (11.1-252.9) | 0.105 | 8 months (0.6-15.4) vs 112 months (0-252.2) | **0.047** | 0.464 | **0.022** |
| CD271+ stroma cells | 24 months (12.3-35.7) vs 132 months (0-290.9) | **0.007** | 8 months (0.8-15.3) vs 112 months (0-244.0) | **0.008** | 0.066 | **0.006** |
| PD-L1+ tumor cells | 34 months (19.2-48.8) vs 36 months (5.4-66.6) | 0.875 | 14 months (0-34.6) vs 13 months (0-34.6) | 0.955 | 0.884 | 1.0 |
| PD-L1+ CTL | 17 months (8.6-25.4) vs 88 months (NA) | **<0.001** | 7 months (4.3-9.7) vs 85 months (NA) | **0.003** | **0.008** | **0.001** |
| CD271+, PD-L1+ cells | 17 months (5.4-28.6) vs 136 months (21.9-250.1) | **<0.001** | 6 months (3.3-8.7) vs 85 months (0-171.2) | **0.002** | **0.005** | **0.006** |

**References**

1. Kaufmann J, Biscio CAN, Bankhead P, Zimmer S, Schmidberger H, Rubak E, et al. Using the R Package Spatstat to Assess Inhibitory Effects of Microregional Hypoxia on the Infiltration of Cancers of the Head and Neck Region by Cytotoxic T Lymphocytes. Cancers (Basel). 2021;13(8):1924.

2. Bankhead P, Loughrey MB, Fernández JA, Dombrowski Y, McArt DG, Dunne PD, et al. QuPath: Open source software for digital pathology image analysis. Scientific Reports. 2017;7(1):16878.

3. Baddeley A, Rubak E, Turner R. Spatial Point Patterns: Methodology and Applications with R: CRC Press; 2015.

4. Therneau TM. A Package for Survival Analysis in R 2022 [R package version 3.4-0:[Available from: <https://CRAN.R-project.org/package=survival>.

5. Uhlén M, Fagerberg L, Hallström BM, Lindskog C, Oksvold P, Mardinoglu A, et al. Proteomics. Tissue-based map of the human proteome. Science. 2015;347(6220):1260419.
